# EARLY NODULIN93 acts via cytochrome c oxidase to alter respiratory ATP production and root growth in plants

**DOI:** 10.1093/plcell/koae242

**Published:** 2024-08-23

**Authors:** Chun Pong Lee, Xuyen H Le, Ryan M R Gawryluk, José A Casaretto, Steven J Rothstein, A Harvey Millar

**Affiliations:** School of Molecular Sciences, University of Western Australia, Crawley, WA 6009, Australia; School of Molecular Sciences, University of Western Australia, Crawley, WA 6009, Australia; Department of Biology, University of Victoria, Victoria, BC V8W 2Y2, Canada; Department of Molecular and Cellular Biology, University of Guelph, Guelph, ON N1G 2W1, Canada; Department of Molecular and Cellular Biology, University of Guelph, Guelph, ON N1G 2W1, Canada; School of Molecular Sciences, University of Western Australia, Crawley, WA 6009, Australia

## Abstract

EARLY NODULIN 93 (ENOD93) has been genetically associated with biological nitrogen fixation in legumes and nitrogen use efficiency in cereals, but its precise function is unknown. We show that hidden Markov models define ENOD93 as a homolog of the N-terminal domain of RESPIRATORY SUPERCOMPLEX FACTOR 2 (RCF2). RCF2 regulates cytochrome oxidase (CIV), influencing the generation of a mitochondrial proton motive force in yeast (*Saccharomyces cerevisiae*). Knockout of ENOD93 in Arabidopsis (*Arabidopsis thaliana*) causes a short root phenotype and early flowering. ENOD93 is associated with a protein complex the size of CIV in mitochondria, but neither CIV abundance nor its activity changed in ruptured organelles of *enod93*. However, a progressive loss of ADP-dependent respiration rate was observed in intact *enod93* mitochondria, which could be recovered in complemented lines. Mitochondrial membrane potential was higher in *enod93* in a CIV-dependent manner, but ATP synthesis and ADP depletion rates progressively decreased. The respiration rate of whole *enod93* seedlings was elevated, and root ADP content was nearly double that in wild type without a change in ATP content. We propose that ENOD93 and HYPOXIA-INDUCED GENE DOMAIN 2 (HIGD2) are the functional equivalent of yeast RCF2 but have remained undiscovered in many eukaryotic lineages because they are encoded by 2 distinct genes.

## Teaser

The plant homolog of a yeast component of the mitochondrial oxidative phosphorylation system plays a role in plant ATP production.

**IN A NUTSHELL**

**Background:** Early nodulins (ENOD) were first discovered 30 y ago as a diverse group of genes expressed at early stages of nodule development in some plants. One of these, ENOD93, is conserved across the plant kingdom and many other eukaryotes. Overexpression of a rice ENOD93 results in higher nitrogen usage efficiency. ENOD93 was predicted to be a membrane protein located in mitochondria. Despite this early discovery, the role of ENOD93 remained to be elucidated.

**Question:** Is ENOD93 needed for mitochondrial respiration? Which part of the respiration process is ENOD93 involved in?

**Findings:** Bioinformatic analysis indicates that ENOD93 is distantly related to the N-terminal part of yeast Respiratory superComplex Factor 2 (RCF2)—a known component of mitochondrial cytochrome c oxidase (complex IV) in yeast. We found out that in Arabidopsis ENOD93 associates with complex IV which plays an essential role in energy production and respiration. By comparing an Arabidopsis mutant, complementation lines, and wild type, we show that losing ENOD93 does not severely affect complex IV activity or abundance but caused a complex IV–dependent progressive loss of respiration rate, affecting mitochondrial membrane potential and cellular ATP production. We provided biochemical and physiological evidence of the role of ENOD93 in plants.

## Introduction

EARLY NODULINS (ENOD) were first discovered 30 y ago as a diverse group of genes expressed at early stages of nodule development in pea (*Pisum sativum*), alfalfa (*Medicago sativa*), and soybean (*Glycine max*; Scheres et al. 1990; Pichon et al. 1992; Kouchi and Hata 1993; Yang et al. 1993). ENOD homologs have subsequently been found in nonleguminous plants (Oldroyd and Downie 2008), and diverse roles in plant biology as membrane proteins promoting the exchange of sugars, amino acids, cofactors, and nutrients have been proposed, with differing levels of supporting evidence in each case (Denancé et al. 2014). One of the most enigmatic of the ENOD set of genes has been ENOD93 for which a molecular function remains unknown. It was first identified in soybean as a gene encoding a 105 amino acid hydrophobic protein rich in alanine and serine residues (Kouchi and Hata 1993). Expression of soybean *ENOD93* during nodulation has subsequently been shown to be controlled by a specific miRNA, and loss of ENOD93 expression is a control point in soybean nodule formation and biological nitrogen fixation (Yan et al. 2015). However, ENOD93-like proteins are not restricted to legumes but are present in nearly all plant genomes and are often present in small gene families. Reddy et al. (Reddy et al. 1998, 1999) identified a series of ENOD93 homologs in rice (*Oryza sativa*). From the rice gene family of 7 members, OsENOD93-1 was prioritized for investigation as a nitrogen use efficiency (NUE) gene candidate because of its strong transcriptional response to both decreasing and increasing N-conditions, suggesting it was a central regulator responding to nitrogen fluctuation (Bi et al. 2007). Transgenic plants overexpressing OsENOD93-1 accumulated amino acids in roots and in xylem sap better than wild type (WT) under low and medium N-conditions and show increased shoot dry biomass and seed yield compared to WT plants under moderate N-conditions (Bi et al. 2009).

ENOD93 is the only member of the ENOD gene set predicted to be located inside mitochondria in plants based on sequence prediction algorithms and subcellular location experiments (Hooper et al. 2014). Expression of a C-terminal fusion of OsENOD93-1 to YFP experimentally confirmed it was located inside mitochondria (Bi et al. 2009). In Arabidopsis (*Arabidopsis thaliana*), a single ENOD93 homolog is found in the nuclear genome encoded by the gene At5g25940. Like its rice counterpart, it is also predicted to be located inside mitochondria (Hooper et al. 2017). AtENOD93 has been found in the Arabidopsis mitochondrial proteome by peptide mass spectrometry in five independent literature reports (Brugière et al. 2004; Heazlewood et al. 2004; Klodmann et al. 2011; Taylor et al. 2011; Senkler et al. 2017). While ENOD93 is only a small 12 kDa protein, it has been observed in native gels at a molecular mass of hundreds of kilodaltons, suggesting ENOD93 is part of a larger protein complex (Klodmann et al. 2011; Senkler et al. 2017).

As the function of ENOD93 is unknown and has eluded determination for decades, we sought to further investigate its sequence similarity, localization within the mitochondria, and molecular function. In so doing, we aimed to determine if the genetic evidence for its role in nitrogen-fixing nodules and in NUE could be reconciled with a role as a conserved mitochondrial membrane protein in plants. We conclude that ENOD93 is a divergent supernumerary subunit of cytochrome c oxidase (CIV). It is related to the N-terminal domain of RESPIRATORY SUPERCOMPLEX FACTOR 2 (RCF2) in yeast and its absence in plants alters the protonmotive force and lowers the rate of oxidative phosphorylation in mitochondria. The high demand for mitochondrial ATP during nitrogen-linked processes may explain its previous links to these processes in plants.

## Results

### ENOD93 sequence conservation among eukaryotes and similarity to the N-terminus of yeast RCF2

ENOD93 sequences have been widely found in sequenced plant genomes and conservation of their core domain is enshrined in the ENOD93 Profam domain (PF03386; Supplementary Fig. S1). Proteins with regions similar to ENOD93 fall into 2 general physicochemical classes. The first class, found in plants, some algae, and some microbial eukaryotes (e.g. amoebozoans), was made up of proteins ∼90 to 130 amino acids in length, typically with 2 predicted transmembrane helices or areas of increased hydrophobicity (Supplementary Fig. S2). Proteins in the other class, found in stramenopiles, haptophytes, and holozoans, were typically longer than 200 amino acids, with 4 predicted transmembrane helices or regions of increased hydrophobicity. In the latter case, homologs generally had an N-terminal region similar to ENOD93 and an additional C-terminal portion containing a conserved “Hig_1_N” domain thought to be involved in the cellular response to hypoxia. The opposite orientation of regions similar to ENOD93 and Hig_1_N was noted in provorans (Tikhonenkov et al. 2022) and bolidophytes.

Blast-based inspection of putative holozoan ENOD93 homologs including the conserved Hig_1_N domain revealed similarity to yeast RCF2 (respiratory supercomplex factor family 2), a protein that plays a role in the regulation of cytochrome c oxidase (complex IV) in the inner mitochondrial membrane (Strogolova et al. 2012). As found in other holozoans and protists with joined ENOD93-like and Hig_1_N regions, yeast RCF2 is composed of a C-terminal portion with 2 transmembrane helices and a Hig_1_N domain, and an N-terminal region lacking obvious similarity to other known protein domains, but that profile hidden Markov model (HMM) searches (Yoon et al. 2009) show to be homologous to ENOD93 (Supplementary Fig. S2).

Given that plant-like ENOD93 homologs are similar only to the N-terminal portion of fungal RCF2, we hypothesized that plant ENOD93s represent separate genes analogous to the N-terminal region of fungal RCF2 and that plants—and microbial eukaryotes with plant-like ENOD93 homologs—likely encode separate and shorter RCF2 proteins that include the Hig_1_N domain. To this end, we identified putative “truncated” Hig_1_N domain-containing RCF2 homologs that are similar to the C-terminal region of fungal RCF2 across plants and in several microbial eukaryotes (Supplementary Figs. S1B and S2B). The Arabidopsis RCF2 candidates, ATHIGD2 (At5g27760) and ATHIGD3 (At3g05550), were already known to possess Hig_1_N domains, and we had previously proposed them as plant-specific components of cytochrome *c* oxidase (Millar et al. 2004). However, they were not recognized at that time to be potential homologs of the C-terminus of RCF2.

In yeast it is known that RCF2 is proteolytically cleaved in vivo into separate N- and C-terminal fragments (Römpler et al. 2016) that are roughly equivalent to the ENOD93-like and C-terminal Hig_1_N regions, suggesting that these proteins may act as physically separate polypeptides even when encoded in a single open reading frame. Yeast encodes an additional mitochondrial protein bearing a Hig_1_N domain, RCF1, which functions in maturation of cytochrome *c* oxidase (Strogolova et al. 2012). RCF1 has an Arabidopsis homolog (Hwang et al. 2017), ATHIGD1 (AT3G48030.1) and is well conserved in terms of distribution and sequence across eukaryotes, including animals. Yeast also encodes another protein, RCF3, that is homologous to the N-terminal region of RCF2 (Römpler et al. 2016). However, plant ENOD93 is more similar to the N-terminal region of yeast RCF2 than to RCF3, and RCF3 homologs could not be identified with confidence outside of Ascomycota and Mucoromycota. This suggests that RCF3 is the product of a more recent gene duplication event within Fungi.

**Figure 1.**
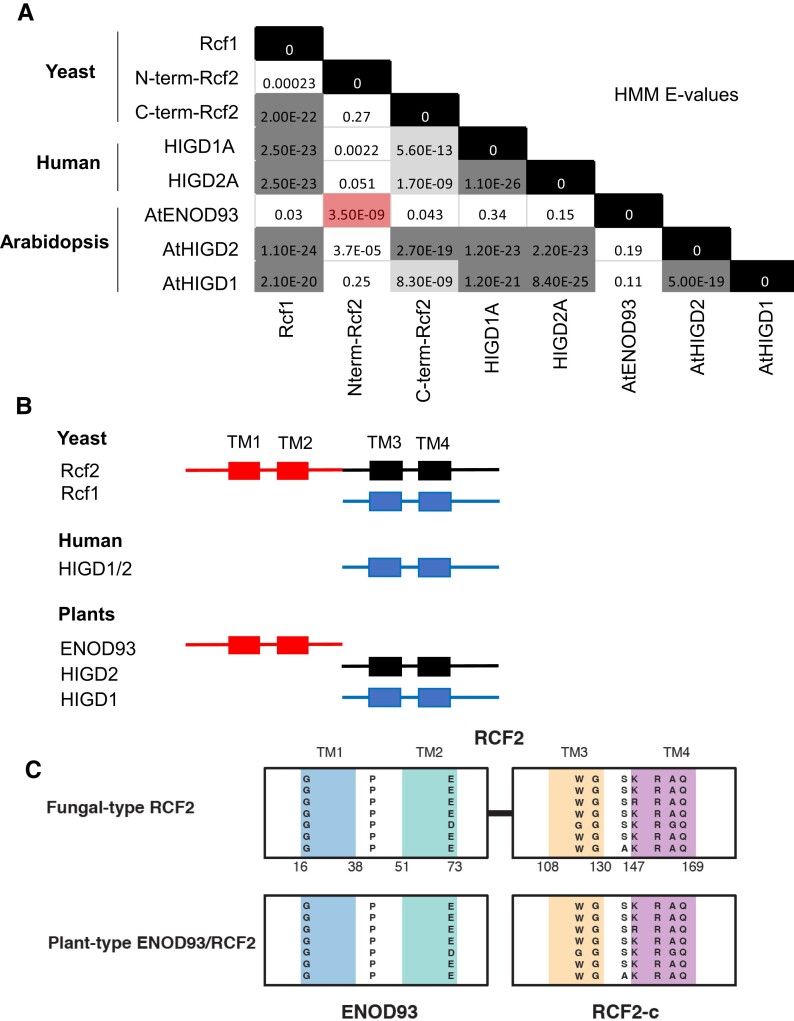
Amino acid sequence similarity between plant, mammal, and yeast RCF2 proteins. **A)** Hidden Markov model (HMM) similarities shown as *E*-values in comparisons of yeast RCF2 N- and C-terminal halves with mammalian HIGD and plant ENOD93 and HIGD proteins. *E*-value shading shows absolute identity (black), high similarity (dark gray), and significant similarity (light gray), and red highlights the significant similarity of ENOD93 and the N-terminal region of Rcf2. **B)** Cartoon of the protein and transmembrane (TM) domain structure of RCF, HIGD, and ENOD proteins and homology of plant, mammal, and yeast proteins with similarity groups shown by three colors. **C)** Similarity of transmembrane sequences in TM1–4 between representative fungal RCF2 and plant-type ENOD93 and RCF2-c-like proteins (named and outlined in more detail in Supplementary Fig. S2) showing single amino acid codes for the highly conserved residues at the borders of and within transmembrane helices. The 4 colors differentiate the 4 different transmembrane domains.

Pairwise profile HMM comparisons show the relative similarity (*E*-value) between Arabidopsis HIG1, HIG2 and ENOD93, yeast RCF1 and RCF2, and human HIGD1A/2A sequences (Fig. 1A). Collectively, these phylogenetic data suggest the existence of separate RCF1-like proteins in plants, yeast, and mammals which contain a Hig_1_N domain, while homologs of the RCF2-like protein in yeast exist as 2 separate genes in plants and algae (Fig. 1B). While the homology of ENOD93 proteins is readily apparent between plants (Pfam PF03386), there are few sequence features conserved broadly among eukaryotes. Nonetheless, there are several highly conserved amino acids, which likely occur near the start or end of transmembrane helices defined for the N-terminal region of yeast RCF2 (Zhou et al. 2021). Sequence similarity of standalone plant-type RCF2 and the fungal-type RCF2 C-terminal region is more apparent, with multiple highly conserved residues at the borders of, and within regions that are transmembrane helices in yeast (Fig. 1C; Supplementary Fig. S2).

### Arabidopsis as a genetic model for ENOD93 functional characterization

**Figure 2.**
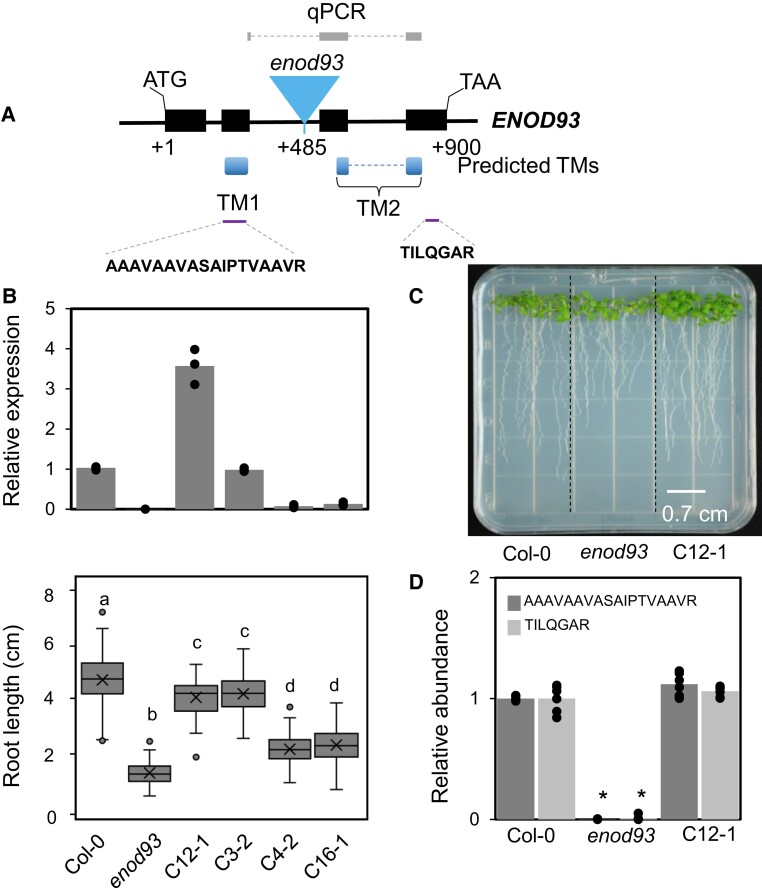
Characterization of enod93 mutant and whole plant phenotype in Arabidopsis. **A)** Genomic structure of *enod93* t-DNA knockout line showing insertion in intronic region (blue triangle) and predicted transmembrane domains based on ARAMEMNON consensus prediction. Black boxes indicate exons, blue boxes indicate transmembrane domains, black lines indicate introns, and dashed lines indicate that introns are spliced out to make final product. **B)** Expression levels of *ENOD93* in different genotypes as determined by qPCR (*n* = 3). Root length of seedlings of those genotypes is shown below with different letters indicating significant differences based on Kruskal–Wallis rank sum test with a post hoc Dunn test (*P* < 0.01, *n* > 50). Center line, median; box limits, upper and lower quartiles; whiskers, 1.5 × interquartile range; points, outliers. **C)** Representative image of vertically grown 7-d-old seedlings of Col-0, *enod93*, and complemented lines on an agar plate under long day condition. **D)** Relative ENOD93 protein abundance in WT, *enod93*, and C12-1 lines based on targeted LCMS of an ENOD93 specific peptides (AAAVAAVASAIPTVAAVR and TILQGAR). Each data point represents mean with overlaid individual data points as dots. Asterisks indicate a significant change as determined by Student's test (*P < 0.05, *n* = 6).

To determine if there is a functional similarity between plant ENOD93 and yeast RCF2, we used the model plant Arabidopsis to perform genetic and biochemical studies. Arabidopsis encodes a single nuclear *ENOD93* gene which co-expresses with many nuclear genes that encode for mitochondrial localized gene products. Of the top 20 most similarly expressed genes (Obayashi et al. 2022), 9 encode mitochondrial-predicted or experimentally mitochondrial-located protein products, and 5 of these are subunits of complex IV, including the aforementioned ATHIGD2 (Supplementary Fig. S3A). To functionally characterize the *ENOD93* gene, an Arabidopsis mutant *enod93* was isolated from the SALK insertion collection. It contains a T-DNA insertion in its second intron (Fig. 2A) that fully disrupts *ENOD93* expression based on primers spanning across the region of the T-DNA insertion (Fig. 2B). Complementation with an *ENOD93* cDNA in the *enod93* background restored expression. The *enod93* mutant line has a short root phenotype that is fully complemented in plant lines expressing the *ENOD93* cDNA at 0.1 to 4 times the level observed in WT (Fig. 2, B and C; Supplementary Fig. S4A). Normal rosette growth was observed in *enod93* and complemented lines at 3, 5, and 6 wk (Supplementary Fig. S4B), but *enod93* developed flower slightly earlier than WT and complemented lines (Supplementary Fig. S4C).

### Localization of ENOD93 within Arabidopsis mitochondria

Previously Arabidopsis ENOD93 had been identified in the mitochondrial proteome isolated from Arabidopsis cell culture and plants (Hooper et al. 2017), and rice OsENOD93-1 had been shown to be mitochondrial localized by YFP fusion studies (Bi et al. 2009). To assess the function of ENOD93, we purified mitochondria from plants of WT, *enod93*, and a complemented line. To confirm that ENOD93 was absent from mitochondria in *enod93,* we developed a targeted mass spectrometry–based MRM assay for 2 ENOD93 peptides in whole mitochondrial extracts. The peptide AAAVAAVASAIPTVAAVR is in the first transmembrane domain before the site of the t-DNA insertion; the peptide TILQGAR is after the second transmembrane domain of ENOD93 after the site of the t-DNA insertion. We showed complete loss of both peptide signals in mitochondrial extracts from *enod93* and recovery to WT levels in the complemented line (Fig. 2D). The abundance of a wide series of other respiratory complex subunits and TCA cycle enzymes were also quantified in the genotypes using previously developed MRM assays for specific peptides, but this showed no statistically significant changes in their abundance between WT, *enod93*, and complemented lines (Supplementary Data Set 1). We also performed native separation of protein complexes by 2D-BN/SDS–PAGE (Supplementary Fig. S4D). Previously, Klodmann et al. (2011) had observed ENOD93 in similar gels as a 12 kDa protein in a large native complex of 250 to 300 kDa and nowhere else in the Arabidopsis mitochondrial proteome map. Native complexome studies from the same group also identified ENOD93 primarily at 250 kDa and found a small proportion in larger respiratory complexes above 1000 kDa and some in the molecular mass region below 100 kDa (Senkler et al. 2017; Supplementary Fig. S3B). These data also clearly differentiate the pattern of complex IV and complex V subunits on BN/PAGE and show ENOD93 clusters with the complex IV pattern. We cut out equivalent protein bands from the 250 to 300 kDa region of our BN/SDS–PAGE gels and used peptide mass spectrometry to confirm these reported localizations (Supplementary Fig. S4D). We identified peptides of ENOD93 in approximately the 250 kDa region alongside complex IV subunits as shown previously. In mitochondria isolated from *enod93*, no ENOD93 peptides were identified in the same position, while we could again detect the presence of peptides for ENOD93 in a complemented line (Supplementary Table S1). This confirmed a colocalization of ENOD93 with respiratory complexes on native gels in WT and complemented line, and no obvious change in the profile of other BN/SDS–PAGE complexes between WT, *enod93*, and the complemented line (Supplementary Fig. S4D).

### Loss of ENOD93 leads to progressive loss of respiratory rate in energized Arabidopsis mitochondria

**Figure 3.**
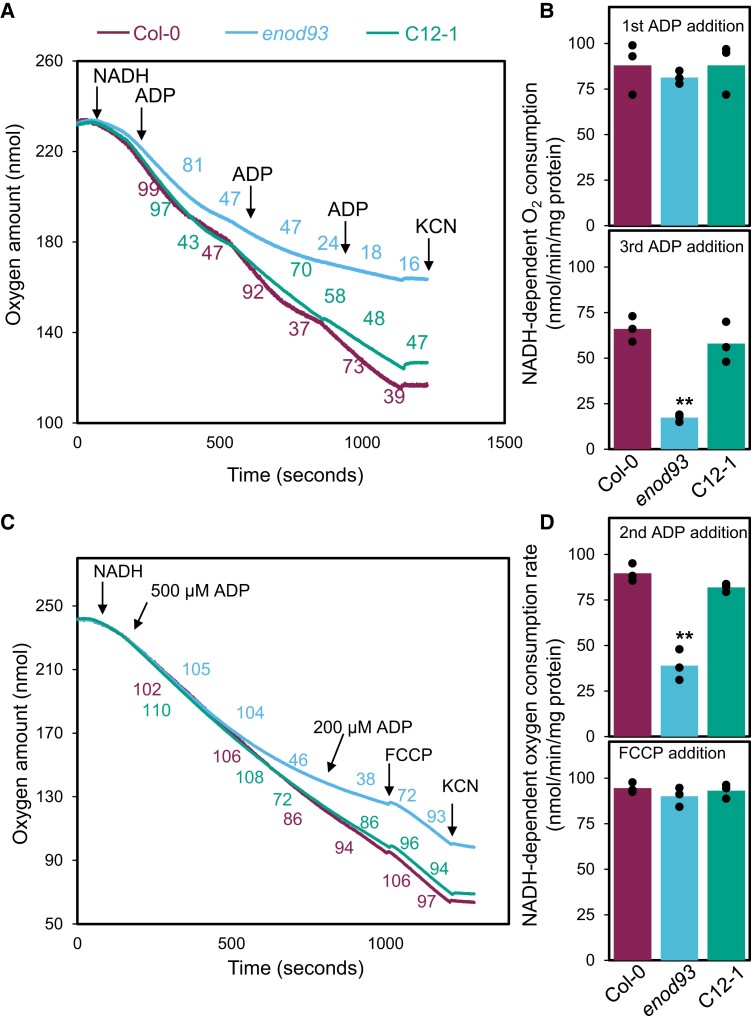
Oxygen consumption characteristics of mitochondria. **A)** Representative trace illustrating NADH-stimulated oxygen consumption by mitochondria purified from Col-0, *enod93*, and C12-1 seedlings (*n* = 1). Oxygen consumption rates in response to the successive treatments are shown in colored numerical values. Substrate additions to all samples are indicated by arrows with the following concentrations: 10 mm NADH, 0.1 mm ADP and 1mm KCN. **B)** State III oxygen consumption rates for purified mitochondria energized with NADH in response to the first (*upper* panel) and third (*lower* panel) ADP addition as indicated in **(A)**. **C)** Representative trace illustrating the “recovery” of oxygen consumption rate by isolated *enod93* mitochondria upon FCCP addition (*n* = 1). Substrate additions are indicated by arrows with the following concentrations: 10 mm NADH, ADP: 0.2 or 0.5 mm ADP, 10 *µ*M FCCP, and 1 mm KCN. **D)** State 3 oxygen consumption rates for purified mitochondria energized with NADH in response to the second ADP addition (upper panel) and to the FCCP addition (lower panel) as indicated in **(C)**. For **B** and **D**, each data point represents mean with overlaid individual data points as dots (*n* = 3). Asterisks indicate a significant change as determined by one-way ANOVA with Tukey post hoc test (*P < 0.05; **P < 0.01).

Substrate-dependent oxygen electrode assays and the addition of cofactors and adenylate nucleotides are typically used to explore functional defects in the mitochondrial respiratory apparatus. We observed a progressive loss of ADP-dependent stimulation of oxygen consumption rate over the course of respiratory assays in mitochondria isolated from *enod93*. During the first addition of ADP and so-called state 3/state 4 transition, *enod93* mitochondria responded similarly to WT. However, following subsequent additions of ADP, stimulation of respiration to the phosphorylating state 3 was not observed (Fig. 3, A and B). This effect was largely reversed in mitochondria from an ENOD93 complemented line. This respiratory effect was independent of the respiratory substrate used in the assay; the same phenomenon was observed in mitochondrial respiration supported by NADH (Fig. 3, A and B), succinate (Supplementary Fig. S5, A and B), or malate and glutamate (Supplementary Fig. S5, C and D). To determine if the respiratory rate effect was reversible, we took *enod93* mitochondria that could no longer be stimulated after multiple ADP additions, recovered them from the assay media by centrifugation, and showed in fresh media that they could regain ADP stimulation and that this could be lost again over time by further ADP incubation (Supplementary Fig. S6). We reasoned this process would abolish the membrane potential of the mitochondria, and it suggested respiratory capacity could be recovered in this manner. To determine if direct loss of membrane potential could indeed reactivate respiratory rate in *enod93* mitochondria, we added the chemical uncoupler FCCP to respiring *enod93* mitochondria after time-dependent ADP inhibition and found recovery of respiratory rate to that observed in WT and the complemented line (Fig. 3, C and D).

### Loss of ENOD93 does not affect respiratory complex abundance or solubilized complex IV activity

**Figure 4.**
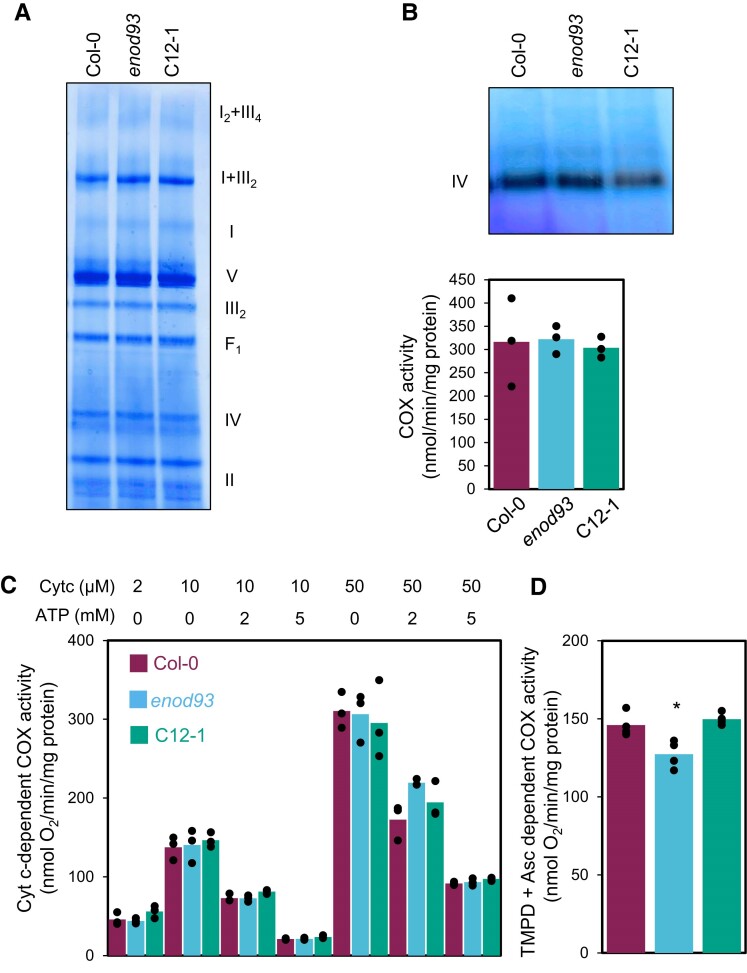
Characterization of mitochondrial respiratory complexes and complex IV activity in isolated plant mitochondria from *enod93*. **A)** Separation of mitochondrial respiratory supercomplexes by 1D-BN/PAGE. Roman numerals correspond to the locations of respiratory complexes. Gels were visualized by Coomassie blue. **B)** visualization of complex IV by activity staining of BN/PAGE and cytochrome c oxidase (COX) activity in hypotonically ruptured, freshly isolated mitochondria in the presence of 50 *µ*M cytochrome c (cyt c). No significant change was found based on one-way ANOVA (*n* = 3, *P* < 0.05). **C)** Sensitivity of cytochrome c oxidase (COX) activity by in hypotonically ruptured, freshly purified mitochondria in the presence of different cytochrome c and ATP concentrations. No significant change was found between genotypes for each treatment based on one-way ANOVA (*n* = 3, *P* < 0.05). **D)** COX activity with N,N,N′,N′-tetramethyl-p-phenylenediamine + ascorbate (TMPD + Asc)as substrates in intact plant mitochondria. All data represent mean with overlaid individual data points as dots (*n* = 3). Asterisks indicate a significant change between Col-0 and *enod93* and between *enod93* and C12-1 as determined by Student's *t*-test (*P < 0.05).

To investigate a basis for these effects within the respiratory chain, we followed up the 2D-BN/PAGE analysis (Supplementary Fig. S4D) by visualizing the abundance of the major electron transport chain protein complexes in ID-BN-native gels but found no differences in their abundance between genotypes (Fig. 4A). BN-native in-gel activity assays of complex IV (Fig. 4B) showed no differences between genotypes, nor did a spectrophotometric assay of complex IV activity in hypotonically ruptured mitochondrial samples (Fig. 4B). As there have been reports of ATP inhibition of complex IV in mammalian mitochondria (Arnold and Kadenbach 1997; Acin-Perez et al. 2011), we directly assessed the effect of ATP on plant complex IV activity (Fig. 4C). This showed a progressive inhibition of complex IV by increasing ATP concentration in WT but no differential effect in *enod93* or the complemented line. A small but statistically significant decrease in complex IV activity could only be observed when TMPD + ascorbate was used to directly deliver electrons to cytochrome c which passes them to complex IV in intact mitochondria (Fig. 4D).

### Loss of ENOD93 slows ATP synthesis and raises the mitochondrial membrane potential

**Figure 5.**
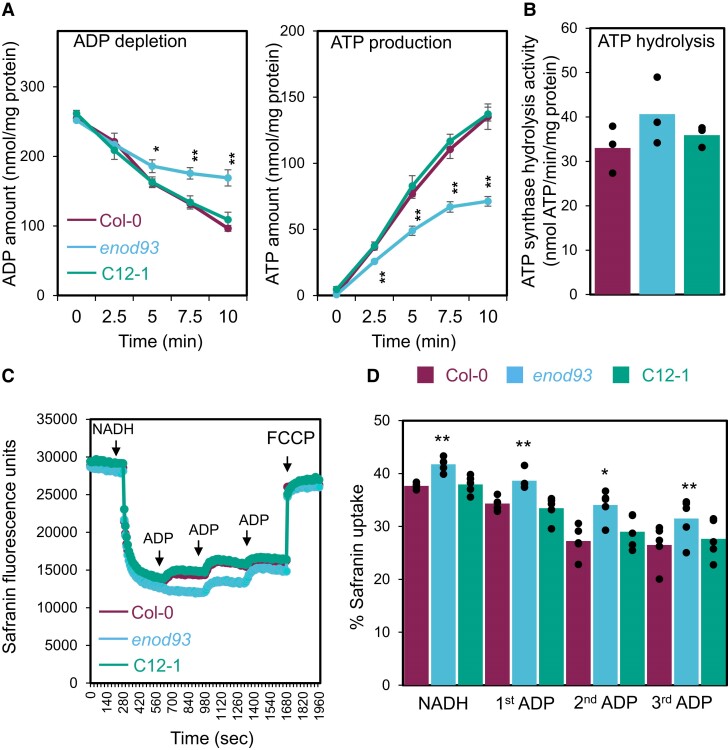
Forward and reverse modes of ATP synthase activity and membrane potential in isolated *enod93* mitochondria. **A)** Time course of ADP depletion (left) and ATP production during NADH-dependent respiration by purified mitochondria. Reaction was initiated after the addition of NADH (10 mm) and ADP (1 mm) to 20 *μ*g of freshly isolated mitochondria and terminated by excess amount of ice-cold 15% TCA solution (*v*/*v*). Following metabolite extraction and solid phase extraction, ADP and ATP concentrations were determined by LC-MS. Each data point represents mean ± S.E. (*n* = 3), and asterisks indicate a significant change between Col-0 and *enod93* and between *enod93* and C12-1 as determined by one-way ANOVA with Tukey post hoc test (*P < 0.05; **P < 0.01). **B)** ATP hydrolysis rate of broken mitochondria. Mitochondrial fraction (20 *μ*g) was subjected to repeated freeze-thaw cycles before 1.5 mm ATP was added to initiate the hydrolysis reaction. ATP hydrolysis rates were determined by monitoring the production of ADP over time using LC-MS. Data represent mean with overlaid individual data points as dots (*n* = 3). No significant change was found based on one-way ANOVA (*P* < 0.05). **C)** Representative trace of safranin fluorescence (*n* = 1) as a measure of membrane potential in mitochondria from WT, *enod93*, and complemented line after multiple ADP additions during NADH-dependent respiration. **D)** Difference in percentage safranin uptake after 1st, 2nd, and 3rd ADP addition in isolated *enod93* mitochondria (mean with overlaid individual data points as dots *n* = 4). Asterisks indicate a significant change between Col-0 and *enod93* and between *enod93* and C12-1 as determined by Student's *t*-test (*P < 0.05; **P < 0.01).

Loss of ADP-stimulated state 3 respiration rate (Fig. 3; Supplementary Fig. S5) suggested a progressive loss of ATP synthesis rate was occurring in *enod93*. To prove this, we directly assayed ATP synthesis and ADP depletion rate in energized mitochondria. We show there was a marked impact in *enod93* mitochondria on the rate of ADP use and ATP generation over time (Fig. 5A). To determine if the reverse rate of ATP hydrolysis by the mitochondrial F_o_F_1_ ATP synthase was also affected, we measured it spectrophotometrically in broken mitochondria and showed it was unaffected in *enod93* (Fig. 5B). Analysis of membrane potential during oxidative phosphorylation using safranin fluorescence showed that *enod93* mitochondria maintained a higher membrane potential throughout respiratory assays than WT or complemented lines, suggesting a restriction of respiratory rate was occurring. In all genotypes, the addition of FCCP depleted the membrane potential (Fig. 5, C and D), and by inference from respiration rate studies (Fig. 3, C and D) can then reactivate complex IV activity. To confirm the importance of this elevated membrane potential on the effect in *enod93* mitochondria, we assessed the effect of malonate addition during succinate-dependent respiration and showed when membrane potential was decreased there was little difference between WT and *enod93* in ADP-dependent oxygen consumption rate (Supplementary Fig. S7, A and B). To show that the operation of complex IV was essential for the elevated membrane potential in *enod93* mitochondria, we repeated the experiment in Fig. 5, C and D using pyruvate and malate as substrate to allow proton translocation via complex I and III but provided ferricyanide as an artificial electron acceptor to bypass complex IV. When complex IV was bypassed in this way, there was no longer any difference in membrane potential between WT, *enod93*, and complemented line mitochondria (Supplementary Fig. S7, C and D).

### Loss of ENOD93 alters oxidative phosphorylation and metabolism in whole plants

**Figure 6.**
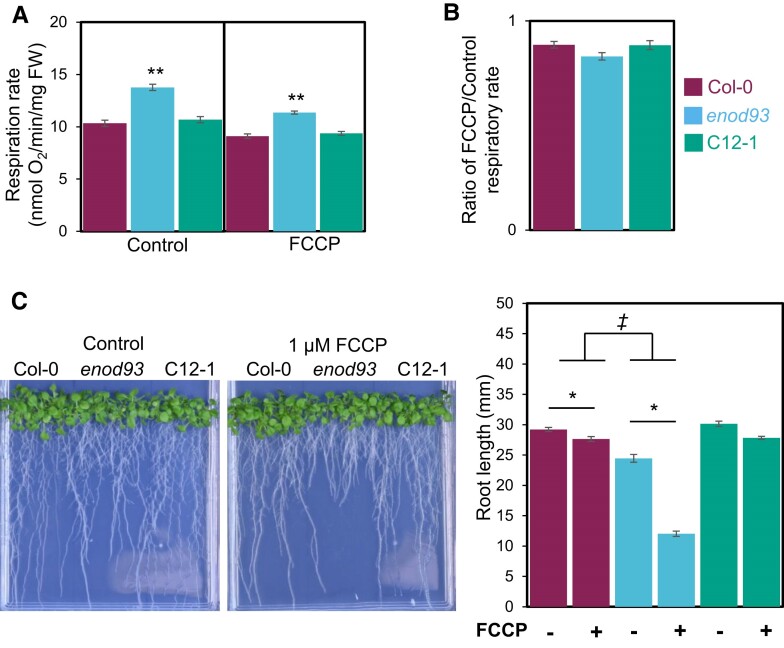
Effect of *enod93* loss and FCCP addition on whole plant respiration and root growth. **A)** Basal respiration rates of 7-d-old Arabidopsis seedlings were measured. FCCP (2 *μ*M) was then added and allowed to equilibrate for 20 min before rates were measured (right). Data represents mean ± S.E. Asterisks indicate a significant change as determined by one-way ANOVA with Tukey post hoc test (**P < 0.01, *n* ≥ 9). **B)** Ratio of basal respiration rates to FCCP-stimulated respiration of seedlings (*n* ≥ 9). No significant change was found based on one-way ANOVA (*P* < 0.05). All measurements were carried out at 25 °C using a Clarke-type oxygen electrode. Data represents mean ± S.E. **C)** Representative image of the effect of FCCP on root growth in *enod93*, Col-0, and C12-1 line. Bar graph of the differences in root length between *enod93* and Col-0. Asterisks represent significant treatment effects; double daggers represent significant genotypic effects (two-way ANOVA; *n* ≥ 17; *P* < 0.01). FW, fresh weight.

To determine if the effect in *enod93* isolated mitochondria could be observed in intact plants, we conducted a series of extra experiments based on our findings. We found whole seedling respiration rate was elevated in *enod93* seedlings but not in an ENOD93 complemented line (Fig. 6A), but there was no difference in respiratory rate responses to the addition of the membrane uncoupler FCCP among genotypes (Fig. 6B). Additionally, we found that the root growth phenotype of *enod93* was more sensitive to FCCP addition to the growth medium than WT or complemented lines, suggesting a longer-term sensitivity in *enod93* seedlings to reduced ATP production rate in intact roots (Fig. 6C).

**Figure 7.**
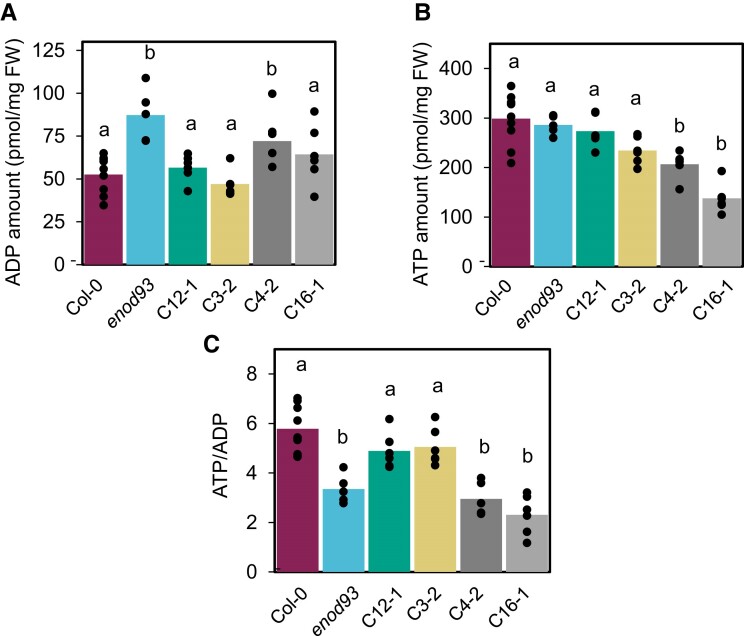
Quantitation of ADP and ATP in roots from WT, enod93, and complemented genotypes. Absolute concentrations of ADP **(A)** and ATP **(B)** in pmol/mg fresh weight (FW) and the corresponding ATP/ADP ratios **(C)** were quantified in roots from a pool of 7-d-old seedlings. Data shown are mean with overlaid individual data points as dots (*n* ≥ 5). Different letters indicating significant differences based on a one-way ANOVA test with a Tukey post hoc test (*P* < 0.05). Col-0, *enod93*, and complemented lines C12-1, C3-2, C4-2, and C16-1 are shown.

To directly assess adenylate levels in whole tissues, root samples from different genotypes were snap-frozen and extracted to measure nucleotide levels. The whole root ADP content was found to be nearly twice as high in *enod93* than WT without a change in root ATP content. Complementation with ENOD93 to different degrees progressively lowered ADP content to WT levels (Fig. 7, A and B). The ATP/ADP ratio approached 6 in WT, lowered to below 4 in *enod93*, recovered in ENOD93 complemented lines with higher expression of *ENOD93,* but did not in complemented lines with lower expression of this transgene (Figs. 2B and 7C).

**Figure 8.**
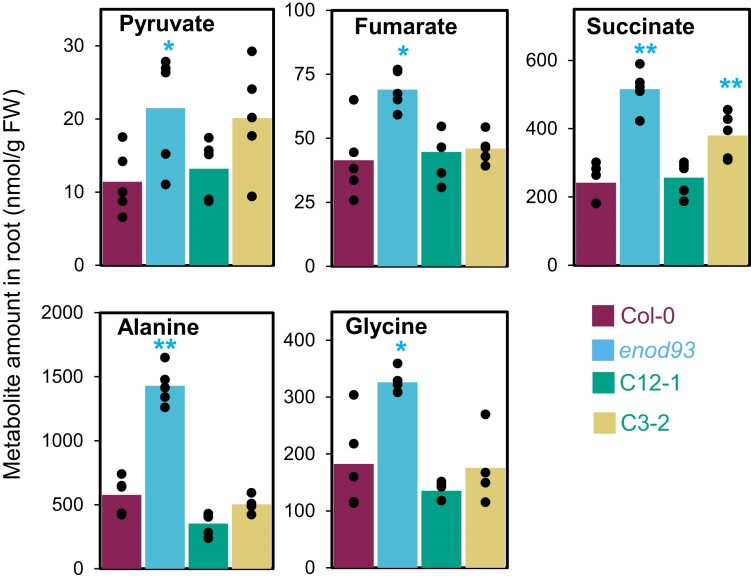
Amino acids and organic acids differing in abundance in roots of different genotypes. Roots excised from a pool of 7-d-old seedlings were collected in liquid nitrogen and extracted metabolites analyzed by LC-MS. Metabolites that demonstrated significant differences between genotype(s) are shown, and the remaining metabolites are shown in Supplementary Table S5. Data from Col-0, *enod93*, and complemented lines C12-1 and C3-2 are shown. Mean values are overlaid with individual data points as dots (*n* ≥ 5) in nmol per gram fresh weight (FW), and asterisks indicate a significant change relative to Col-0 as determined by Student's *t*-test (*P < 0.05; **P < 0.01).

A selection of the Arabidopsis genotypes with altered energetic states (Fig. 7) was also profiled for their absolute abundance of a range of amino acids and organic acids in roots and leaves. These data showed elevation of pyruvate, alanine, glycine, fumarate, and succinate levels in *enod93* roots, compared to WT levels, and recovery of the same organic and amino acid levels in complemented lines (Fig. 8; Supplementary Data Set 2). In contrast, there were very few and only minor differences in amino acid and organic acid abundances in leaves of *enod93* (Supplementary Data Set 2).

## Discussion

ENOD93 is a small, ubiquitously expressed plant membrane protein with an unknown protein domain that has evaded functional evaluation for decades (Kouchi and Hata 1993; Reddy et al. 1998; Bi et al. 2009; Yan et al. 2015). In its absence, we show that plant mitochondria suffer a progressive loss of function which to our knowledge has never been reported for any other mutant of an electron transport chain component in plant mitochondria. We also show that adenylate levels and metabolic pathways are perturbed in vivo in the absence of ENOD93 leading to reduced root growth. Combining evidence from genetics, mitochondrial biochemistry, and phylogenetic sequence analysis, we hypothesize that ENOD93 plays a widespread and ancient role in ensuring the function of the mitochondrial electron transport chain by influencing the function of complex IV and through it the rate of ATP synthesis in energized mitochondria.

### ENOD93 affects plant mitochondria function and is associated with complex IV

Loss of ENOD93 has a profound impact on respiratory function and ATP synthesis in Arabidopsis, however, precisely explaining the mechanism of this phenomenon remains complex. We show this effect is independent of the respiratory substrate used in the oxygen consumption assays, indicating the lesion is downstream of respiratory substrate dehydrogenases. We observe the effect of ENOD93 loss on total CIV activity is minor, only becoming evident when it is operating as part of the respiratory chain in energized mitochondria. The loss of ENOD93 slows the oxygen consumption rate to a much greater degree when the membrane potential has increased to a certain level, and this is then linked to a marked decrease in ATP synthesis rate. The importance of a high membrane potential in the reversible inactivation of complex IV and ATP synthesis in *enod93* in underlined by the 2 scenarios that appear to lead to recovery. Firstly, we show centrifugal recovery of mitochondria and re-assaying of oxygen consumption that removes respiratory substrates and de-energize the membrane allows recovery (Supplementary Fig. S6), and secondly, we evidence that addition of FCCP that will dissipate the inner membrane electrical potential recovers full complex IV activity (Fig. 3).

The evidence for the primary link of ENOD93 to the electron transport chain being via complex IV comes from a series of published proteomics data, the case of the related RCF2 protein from yeast, and the information provided in this report. Gel-spot identifications from BN/PAGE separations of plant mitochondria first observed a native complex of ∼250 kDa that contained peptides for ENOD93 (Klodmann et al. 2011) and also showed that HIG2 is associated with CIV (Millar et al. 2004). Subsequently, complexome profiling of similar gels tracked quantitative changes in ENOD93 abundance and again found it in a mass range equivalent to other CIV subunits (Senkler et al. 2017), but this association was not claimed at the time due to the proportion of ENOD93 being found in a dissociated state toward the bottom of BN/PAGE separations. Re-clustering of these data now shows that both ENOD93 and HIG2 are in the protein complex with CIV subunits and that the ENOD93 in lower native mass complexes co-migrates with Cox6b (Supplementary Fig. S3B). The mitochondrial complexome data from Senkler et al. (2017) also argue against association of ENOD93 with the pattern of complexes and subcomplexes of F_o_F_1_ATP synthase subunits (Supplementary Fig. S3B). In yeast, RCF2 association has mostly been studied by BN/PAGE studies (e.g. Strogolova et al. 2012, Römpler et al. 2016; Strogolova et al. 2019) and was not found in early crystal structures of CIV. But more recent CryoEM CIII/CIV super complex structures (Hartley et al. 2020; Moe et al. 2023) contain the C-terminal HIG domain of RCF2 bound to Cox12 (which is the yeast equivalent of COX6b in plants). CryoEM of the plant complex IV from mung bean did not identify HIG2 or ENOD93 in the structure but did observe HIG2 by mass spectrometry in samples used for the structural determination (Maldonado et al. 2021); consistent with the instability of this association. The RCF2 association with the CIV2/CIII supercomplex in CryoEM structures is far away from the CIV/CIII interface, suggesting it is definitively with each CIV molecule separately. Collectively this information is consistent with HIG2 and ENOD93 both being associated with intact complex IV in plants and ENOD93 dissociating from the complex in detergent preparations in association with Cox6b, providing a putative site of complex IV interaction. There are several hypotheses to explain the apparent association of ENOD93 with complex IV but a major effect on ATP synthesis. On the one hand, the mechanics of supercomplex functions are not fully understood, so complex IV super-associations with complex V in vivo may facilitate the effect observed. On the other hand, heterogeneity in mammalian and yeast mitochondrial membranes show laterally separated complex IV and complex V in cristae that are reported to establish non-homogenous kinetics of oxidative phosphorylation between them (Toth et al. 2020; Rieger et al. 2014). Further work is required to characterize heterogeneity in electrical and pH gradients in plant mitochondria and in vivo complex IV super-associations to further explore these possibilities.

### ENOD93 is similar to the N-terminus of RCF2 and they share a degree of functional equivalence

Evaluating the evidence for functional equivalence of RCF2 and ENOD93 is complicated because ENOD93 is only homologous to the N-terminal domain of RCF2. Much of the evidence for the functional role of RCF2 in yeast arises from a study of Δrcf1, Δrcf2, or Δrcf1rcf2 mutants. Loss of RCF1 decreases the abundance of complex IV, and it appears to have a role in CIV assembly through binding to COX3 (Strogolova et al. 2012). In contrast, loss of RCF2 does not change complex IV abundance, but alters the activity of the enzyme (Strogolova et al. 2019). Complementation studies in yeast show that RCF1 and RCF2 are not functionally redundant (Römpler et al. 2016; Homberg et al. 2021). Yeast mitochondria lacking both RCF proteins have lower mitochondrial membrane potential and the respiratory chain becomes inactive over time (Strogolova et al. 2019). In mutants lacking both RCF1 and RCF2 proteins, there is a proton leak back through complex IV, suggesting that the HIG domains of RCFs might be involved in preventing proton leak from the IMS to the matrix through complex IV (Hoang et al. 2019). Recently it was also found that fusing the RCF2 N-terminus to RCF1 could rescue an Δ*rcf2Δrcf3* mutant (Homberg et al. 2021) which was surprising as previously the focus had been only on the RCF2 C-terminal HIG domain as having unique capabilities in the regulation of complex IV activity (Römpler et al. 2016).

Based on our phylogenetic and functional evidence, we propose that RCF2 in yeast is a fusion of 2 genes found in other eukaryotes that may have related but different functions, which complicates the interpretation of joint loss experiments and the chimeric forms of RCF2 that have been generated and studied. Independent function of the RCF2-N terminal and RCF2-C terminal is certainly possible given that yeast RCF2 can be proteolytically cleaved inside mitochondria into these 2 parts, allowing its 2 halves to function independently at a biochemical level (Römpler et al. 2016). Collectively, (i) the functional evidence that yeast RCF2 functions in CIV activity regulation associated with the protonmotive force and the related observations in *enod93* plants, (ii) the physical association of RCF2 C-terminus with COX12 in the CryoEM structure and the co-migration of ENOD93 and COX6b in plants, and (iii) the profile hidden Markov model link between the RCF2 N-terminus and ENOD93 in phylogenetic studies across eukaryote provide a substantial basis for suggesting a functional equivalence between these 2 proteins. In addition, at the whole cell level, Δrcf2 elevated whole yeast cell respiration rate (Strogolova et al. 2019) as observed in *enod93* plants (Fig. 6A), and yeast Δrcf2 cells were more sensitive to the K+/H+ ionophore nigericin (Strogolova et al. 2019) as *enod93* plants were to the uncoupler FCCP (Fig. 6B). One notable difference is that the progressive respiratory inhibition in *enod93* in isolated plant mitochondria appears to be fully reversible by membrane de-energization, while the progressive respiratory inhibition observed in yeast Δrcf1Δrcf2 is considered to be an irreversible complex IV suicide inactivation requiring resynthesis of the enzyme (Hoang et al. 2019). This difference may underlie distinct roles of the N- and C-terminal domains of RCF2 that have yet to be functionally evaluated in yeast by deleting only the N-terminus of RCF2 and studying mitochondrial functions. The loss of ENOD93 in Arabidopsis did not affect the abundance HIGD2 the homolog for the C-terminus of RCF2 (Supplementary Data Set 1), potentially supporting the milder phenotype compared to the more severe decline in complex IV activity in yeast.

### Reconciling ENOD93 function with reported links to nitrogen fixation and nitrogen metabolism in plants

ENOD93s were previously identified in plants as genes with elevated expression in N_2_ fixing root nodules and/or highly responsive to plant nitrogen status (Kouchi and Hata 1993; Bi et al. 2009; Cabeza et al. 2014; Yan et al. 2015). In N_2_ fixing root nodules, mitochondria in infected cells operate at a low oxygen concentration and an elevated membrane potential to generate high rates of ATP synthesis to sustain the symbiosis (Millar et al. 1995). Based on our discovery of ENOD93 function, the nodule enhanced expression of ENOD93, and the ability of miR393j-3p to regulate it and influence nodule formation in soybean (Yan et al. 2015) can be reinterpreted as a biochemical effect of this gene on ATP availability. Cabeza et al. also showed there is a strong association between nitrate inhibition of nodule N_2_ fixation in Medicago and downregulation of *ENOD93* (Cabeza et al. 2014). Close inspection of their supplemental transcript profiling data also shows this effect is associated with downregulation of other complex IV subunit genes, suggesting mitochondrial inhibition of complex IV is responsible. The biotechnological use of ENOD93 to alter NUE in rice (Bi et al. 2009) is consistent with the fact that nitrate uptake and assimilation both use a substantial proportion of cytosolic ATP in plants (Glass 2003; Xu et al. 2012). The specific cost for net NO_3_^−^ uptake ranges from 3 to 5 ATP in plant species growing at different rates (Scheurwater et al. 1999). Following reduction to ammonia, assimilation of N into amino acids also has a substantial additional ATP cost via glutamine synthase (GS) and asparagine synthase (ASN). The apparent N control of ENOD93 expression could be hypothesized to enable plants to modulate ATP synthesis rate to sustain N uptake and assimilation of amino acids and provide the high ATP demand needed in N_2_ fixing root nodules. This hypothesis would need to be directly assessed in N_2_ fixing root nodules or in NUE studies of ENOD93 overexpression.

### ENOD93 is an ancient and widely distributed component of mitochondria

The phylogenetic distribution of ENOD93 across green plants and in iterative searches of the NCBI nr database using psi-blast showed this domain is very widely observed in many eukaryotic lineages (Supplementary Fig. S8). ENOD93 homologs, can be consistently identified in all major groups interrogated, including green algae, and early-diverging plant lineages (e.g. hornworts, mosses, lycophytes, and ferns), along with diverse gymnosperms and angiosperms. Using a profile Hidden Markov model based on green plant ENOD93 to interrogate protein sequences from diverse microbial eukaryotes, we were also able to identify putative ENOD93 homologs in multiple non-plant eukaryotic lineages, including cryptophytes, amoebozoans, collodictyonids, apusomonads, diverse stramenopiles, haptophytes, and obazoans (i.e. fungi and unicellular relatives of fungi and animals, but not animals themselves), though homologs were not identified in many other groups (Supplementary Fig. S8). Although functional annotation is not available for any of these putative ENOD93 homologs, candidate homologs from amoebozoans *Acanthamoeba castellanii* and *Dictyostelium discoideum* have been identified in highly purified mitochondria (Gawryluk et al. 2014; Freitas et al. 2022). This patchy but very wide distribution of putative ENOD93 homologs across diverse lineages suggests its ancient origins within eukaryotes. The uniting of functional evidence for its role in plants and yeast shown here provides a strong foundation for its wider evaluation across eukaryotes as an important factor in electron transport chain regulation that is capable of altering mitochondrial ATP synthesis rate.

## Materials and methods

### Homology detection

To investigate the distribution of ENOD93 homologs across land plants and green algae, the Arabidopsis (*A. thaliana*) homolog (AT5G25940.1) was used as a query in iterative psi-blast searches of the NCBI nr database (Altschul et al. 1997). After each iteration, evident ENOD93 homologs from diverse lineages, including charophytes, zygnematophytes, liverworts, hornworts, mosses, ferns, lycophytes, and angiosperms, were manually selected to refine the position-specific scoring matrix in subsequent searches. Putative ENOD93 homologs were identified in other eukaryotic groups using profile Hidden Markov model (HMM) searches of phylogenetically broad protein sequence datasets including MMETSP (Keeling et al. 2014) and PhyloFisher (Tice et al. 2021), along with select individual datasets. Initial searches were performed using Hmmer v3.3.2 based on multiple alignments of diverse plant and green algal ENOD93 homologs generated using MAFFT v7.475 under the L-INS-i iterative refinement method (Rozewicki et al. 2019). Where likely homologs of ENOD93 were identified in other eukaryotes, those homologs were added to the initial alignment, from which a broader HMM was generated and again used to iteratively query the datasets described above. Significant matches were defined as those that met the default Hmmer inclusion threshold and reuse. In order to increase our confidence in assigning plant ENOD93 and Rcf2 proteins as *bona fide* homologs of the fungal RCF2 N- and C-terminal regions, respectively, we also tested the reciprocal HMM search, with the expectation that fungal RCF2 queries retrieve homologs of both plant ENOD93 and RCF2. Briefly, homologs of RCF2 from diverse fungi were collected and aligned as above and used to generate profile HMMs and to query plant datasets. Phylogenetically broad bioinformatic searches for homologs of RCF1 and RCF3 were carried out essentially as above, though the high conservation of RCF1 precluded the need for HMM searches. Prediction of transmembrane helices was done with DeepTMHMM v1.0.19 (Hallgren et al. 2022). However, even known transmembrane helices (e.g. in yeast RCF2) were not predicted accurately. In cases where the expected number of transmembrane helices was not predicted, Kyte-Doolittle hydropathy plots were manually inspected for putative ENOD93 and RCF2 sequences to verify their physicochemical plausibility as homologs. The *E*-values for HMM pairwise comparisons shown in Fig. 1B were generated using HHpred (Zimmermann et al. 2018).

### Plant material and growing conditions

A seed stock of an Arabidopsis T-DNA insertion mutant line (SALK_204202; in Columbia-0 background, and herein *atenod93*) was identified and obtained from the Arabidopsis Biological Resource Center (ABRC). When planted on soil, seeds were stratified for 48 h and then spread on soil. Plants were grown in a growth chamber maintained under a 16-h photoperiod (100 to 150 *μ*mol m^−2^ s^−1^) at 21 °C during the day and 18 °C during the dark, with 60% relative humidity. Flowering time was recorded as the date of first visible flower bud appearance. For all in vitro experiments, plants were grown on agar plates using 1% agar (*w*/*v*) supplemented with ½-strength Murashige & Skoog (MS) basal salts (PhytoTech Labs) at pH 5.7, 1% sucrose (*w*/*v*), and without additional nitrogen sources. Seeds were sterilized by shaking them for 10 min in 70% ethanol (*v*/*v*) and 0.05% Triton-X (*v*/*v*). The solution was removed and seeds were allowed to sit for 5 min in 100% ethanol (*v*/*v*) and then placed in fresh ethanol for an additional 3 min. Seeds were allowed to dry on sterile filter paper in a flow hood and kept in sterile microfuge tubes. After placing them on plates, they were allowed to stratify at 4 °C in the dark for 48 h. Unless stated otherwise, plates were grown vertically under a 16-h photoperiod as described above. To isolate mitochondria, surface-sterilized seeds were grown in ½-strength MS medium, 2 mm MES pH 5.7) supplemented with 1% (*w*/*v*) sucrose and 0.1% (*w*/*v*) agar for 14 to 16 d with gentle agitation (40 to 60 rpm) under long day conditions. For FCCP sensitivity of root phenotypes, seeds are surface sterilized and stratified as described above. Arabidopsis seedlings were grown for 2 wk on vertical ½-strength Murashige and Skoog agar (10 g·L^−1^ agar, 10 g·L^−1^ sucrose, 0.4 g·L^–1^ MES) plates with and without 1 *µ*M FCCP added before autoclaving. 20 mm FCCP stock was made in 100% ethanol (v/v). Root lengths were measured and analyzed using ImageJ 1.52v.

### Generation of AtENOD93 complementation lines

The nucleotide sequence of the *A. thaliana ENOD93* gene was retrieved from the NCBI database with the GenBank ID At5g25940. The full-length coding region of AtENOD93 was amplified from Arabidopsis cDNA using the primer pairs AtENOD93-GFwd and AtENOD93-GRev to generate a construct for overexpression in the *atenod93* mutant background employing the Gateway technology with pDONR 221 as the entry vector and pB2GW7 as the destination vector. The resultant 35S:AtENOD93 construct was amplified in *E. coli* DH10b cells and then transferred to *Agrobacterium tumefaciens* strain LBA4404 which was used to transform mutant plants via the standard floral dip method (Clough and Bent 1998) and screened with 0.1% solution (*v*/*v*) of the herbicide Basta (glufosinate; Bayer CropScience). Positive transgenic lines were grown to T2 and T3 generations to obtain homozygous seeds and seeds from the T3 generation were used in subsequent studies.

### Genotyping of the transgenic and T-DNA insertion lines

The putative transgenic plants that survived the herbicide treatment were confirmed for the integration of the transgenes by PCR using genomic DNA as a template. DNA samples were obtained using the TPS extraction method (Edwards et al. 1991). The PCRs were run with Platinum Taq DNA Polymerase (Life Technologies) and using a vector-specific primer (pB2GW7-Fwd) and a promoter-specific (35S-Fwd) primer, either combined with the transgene-specific primer AtE93-Rev (listed in Supplementary Table S2).

For verifying the T-DNA insertion site in *atenod93*, lines were genotyped using primers designed via SALK's iSect Primer program (atenod93-LP, atenod93-RP, and LBb1.3). To sequence the left border of the T-DNA insert and the adjacent genomic sequence, the region was first amplified using the primers TDNA-SeqLB-R and LBb1.3. The resulting amplicon was then sequenced using the same primers used for the amplification. All primers used for cloning and genotyping are listed in Supplementary Table S2.

### Quantitative RNA analysis

To analyze the expression of *AtENOD93*, total RNA was isolated from 100 mg of rosette leaf tissue from WT, *atenod93*, overexpression, and complementation lines using the Total Plant/Fungal RNA Isolation Kit (Norgen Biotek Corp). After removing any contaminating DNA, 1 *µ*g of the total RNA from each sample was reverse transcribed into cDNA using qScript cDNA SuperMix (Quanta Biosciences) according to the manufacturer's protocol. qPCR was performed in a 20 *µ*L reaction using 50 to 100 ng of cDNA from each sample and the PerfeCTa SYBR Green SuperMix (Quanta Biosciences) on the ABI7300 system (Applied Biosystems). The PCR program adjusted for the amplification of the target fragment consisted of 1 cycle at 95 °C for 2 min, and 40 cycles of 95 °C for 15 s and 60 °C for 30 s. The specific primers AtENOD93-qFwd and AtENOD93-qRev were designed with the software Primer3 and used to amplify *AtENOD93* cDNA. Relative expression values from 2 technical and 3 biological replicates were calculated using Applied Biosystem's included software, which uses the 2^−ΔΔCT^ method. The *Actin7* gene was used as the internal reference for normalization. Primers used in expression analysis are listed in Supplementary Table S2.

### Isolation of mitochondria, and measurements of oxygen consumption, ATP synthesis, and membrane potential

Mitochondria were isolated from 2-wk-old Arabidopsis seedlings as described previously (Sweetlove et al. 2007). Substrate-dependent O_2_ consumption by purified mitochondria was measured in a computer-controlled Clark-type O2 electrode unit according to Lee et al. (Lee et al. 2010), using 1 mL of respiration medium (0.3 m sucrose, 5 mm KH_2_PO_4_, 10 mm TES, 10 mm NaCl, 4 mm MgSO_4_, 0.1% (w/v) BSA, pH 7.2) and 100 *μ*g of mitochondrial sample. Cytochrome c oxidase activity was determined by the rate of oxygen consumption (Neuburger et al. 1982), except different concentrations of exogenous cytochrome c and ATP were added. For measurement of O_2_ consumption by cytochrome c oxidase activity with endogenous cytochrome c, 300 *µ*M N,N,N′,N′-tetramethyl-p-phenylenediamine dihydrochloride (TMPD) plus 10 mm ascorbate were added.

Substrate-dependent forward mode of ATP synthase activity (i.e. ATP production) was determined by incubating 20 *µ*g freshly isolated mitochondria in 200 *µ*L respiration medium with 0.1 mm NADH and 1 mm ADP. The reaction was terminated at the specified time by adding 800 *µ*L 15% trichloroacetic acid (*v*/*v*) and snap-freezing in liquid nitrogen. For the reverse mode of ATP synthase activity (i.e. ATP hydrolysis), isolated mitochondrial fractions were subjected to at least 3 freeze-thaw cycles in liquid nitrogen. Following resuspension in respiration buffer, mitochondria (20 *µ*g in 200 *µ*L) were incubated in 1 mm ATP. At the specified time, the reaction was stopped by adding 800 *µ*L 15% trichloroacetic acid and snap-freezing in liquid nitrogen. ADP and ATP concentrations in these samples were determined by mass spectrometry described below.

Safranin O was used as a membrane-permeable cation that crosses to the matrix surface of the inner mitochondrial membrane in proportion to the density of negative charge. Membrane potential across the inner mitochondrial membrane was determined by the decrease in safranin O fluorescence. For each assay, 20 *µ*g freshly isolated mitochondria were incubated in 200 *µ*L respiration medium containing 1 *µ*M safranin O. Mitochondria were equilibrated for 5 min before state 2 respiration was initiated by respiratory substrate addition. State 3 respiration was established by multiple additions to the final concentration of 1 mm ADP. The reaction was terminated by adding 4 *µ*M FCCP to dissipate ΔΨ to zero. Safranin O fluorescence was recorded using FLUOstar Omega plate reader (excitation at 544 nm and emission at 590 to 10 nm). The percent safranin uptake at any time point was calculated as (micromolar safranin at 0 ΔΨ − micromolar safranin at time x)/micromolar safranin at 0 ΔΨ.

### Blue native electrophoresis and protein identification by liquid chromatography tandem mass spectrometry (LC-MS/MS)

Separation of digitonin-solubilized mitochondrial proteins on 1D-BN/PAGE and 2D-BN/SDS–PAGE was performed (Eubel et al. 2003), using a 4.5% to 16% gradient (*v*/*v*). In-gel staining of complex IV activity was carried out as outlined previously (Sabar et al. 2005).

Gel spots were excised from 2D-BN/SDS–PAGE and sliced into smaller cubes (∼1 mm) with a razor blade where necessary. Peptides in each gel piece were digested overnight with trypsin at 37 °C and then extracted as previously described (Nelson et al. 2014). Following resuspension of digested peptides in 20 *μ*L of 2% (*v*/*v*) acetonitrile and 0.1% (*v*/*v*) formic acid, samples were filtered before they were loaded onto a C18 high-capacity nano LC chip (Agilent Technologies) and eluted into Agilent 6550 Q-TOF with a 1200 series capillary pump as described previously (Nelson et al. 2014).

Results were searched against an in-house Arabidopsis database comprising ATH1.pep (release 10) from The Arabidopsis Information Resource (TAIR) and the Arabidopsis mitochondrial and plastid protein sets (the combined database contained a total of 33,621 protein sequences with 13,487,170 residues) using the Mascot search engine version 2.3. Search parameters include error tolerances of 20 ppm for MS and 0.5 D for MS/MS, “max missed cleavages” set to 1, and variable modifications of oxidation (Met) and carbamidomethyl (Cys). Results were filtered with an ion cutoff score of 0.

### Analysis of mitochondrial peptides by liquid chromatography selective reaction monitoring mass spectrometry (LC–SRM–MS)

One hundred micrograms of mitochondrial protein were precipitated in 100% acetone overnight at −20 °C, and the pellets were washed with ice-cold acetone for 3 times. Samples were alkylated, trypsin digested, desalted, and cleaned as previously described (Petereit et al. 2020). The digested peptides were loaded onto an AdvanceBio Peptide Map column (2.1 mm × 250 mm, 2.7  *μ*m particle size; part number 651750-902, Agilent), using a Thermo UltiMate 3000 RSLCnano System coupled to an Thermo Altis Triple Quadrupole MS. The column was heated to 55 °C, and the column flow rate was 0.4 mL/min. The binary elution gradient for HPLC was described previously (Le et al. 2022). The list of peptide transitions used for SRM–MS is provided in Supplementary Table S2 with peak area data. Peak area of targeted peptides was determined using the Skyline software package version 21.2.0.425. Peptide abundances from each sample were normalized against voltage-dependent anion channel (VDAC).

### Analyses of organic acids and amino acids by LC–SRM–MS

Leaf discs or roots (∼25 mg) were collected at specified time points and immediately snap-frozen in liquid nitrogen. Metabolites were extracted as previously specified (Lee et al. 2021).

For LC-MS analysis of organic acids, sample derivatization was carried out based on a previously published method with modifications (Han et al. 2013). Samples were analyzed by an Agilent 1100 HPLC system coupled to an Agilent 6430 Triple Quadrupole (QQQ) mass spectrometer equipped with an electrospray ion source as described previously (Lee et al. 2021).

For amino acid quantification, dried samples were resuspended in 50 mL water as described previously (Le et al. 2021). Briefly, chromatographic separation was performed using Agilent Poroshell 120 HILIC-Z column, using mobile phases of 20 mm ammonium formate in water (solvent A) and 20 mm ammonium formate in acetonitrile (Solvent B). The elution gradient (*v*/*v*) was 100% B at 0 min, 92% B at 1 min, 70% B at 10 min, 30% B at 10.5 min, 30% B at 12.5 min, 100% B at 12.5 min and 100% B at 25 min. The column flow rate was 0.4 mL/min; the column temperature was 35 °C, and the autosampler was kept at 10 °C. The Agilent 6430 QQQ-MS was operated in positive ion mode in SRM mode. Data acquisition and LC-MS control were done using the Agilent MassHunter Data Acquisition software (version B06.00 Build 6.0.6025.4). The autosampler was kept at 10 °C. The QQQ-MS was operated in SRM mode using the following operation settings: capillary voltage, 4,000 V; drying N_2_ gas and temperature, 11 L/min and 125 °C respectively; and nebulizer, 15 psi. Data analysis was carried out using MassHunter Quantitative Analysis Software (version 10.1, Build 10.1.733.0). Metabolites were quantified by comparing the integrated peak area with a calibration curve obtained using authentic standards and normalized against fresh weight and internal standards.

### Analyses of nucleotides by LC–SRM–MS

Absolute quantitation of ADP and ATP by LC-MS was carried out according to previous reports (Straube et al. 2021) with slight modifications. Briefly, ∼25 mg roots or 50 mg leaf discs were collected and immediately snap-frozen in liquid nitrogen. Samples were ground to a fine powder and 1 mL of ice-cold 15% TCA solution (v/v) supplemented with ^13^C_5_,^15^N_5_-AMP as an internal standard. Following centrifugation at 24,000 × *g* for 10 min (4 °C), 1 mL 78/22 DCM/TOA was added to the supernatant. The mixture was then vortexed and centrifuged at 5,000 × *g* for 2 min. The upper phase was collected and diluted in 1 mL H_2_O and 5 *µ*L 0.5% acetic acid (*v*/*v*). The resulting mixture was applied to a Strata X-AW SPE cartridge (pre-equilibrated with 1 mL methanol, 1 mL 2/25/73 formic acid/methanol/H_2_O, and 1 mL 10 mm ammonium acetate pH 4.5) and the flow-through was discarded. The cartridge was then washed with 1 mL 1 mm ammonium acetate (pH 4.5) and 1 mL methanol before nucleotides were eluted with 0.5 mL 20/80 ammonia/methanol twice. The eluate was transferred to a new tube and dried using a SpeedVac.

Dried samples were resuspended in 100 *µ*L water. Chromatographic separation was performed using Agilent Poroshell 120 HILIC-Z column, using mobile phases of 5 mm ammonium acetate pH 9.0 (solvent A)/5 mm ammonium acetate, 90% ACN (Solvent B, v/v). Solvents A and B were supplied with 0.1% (*v*/*v*) Infinity Lab deactivator additive (Agilent) to improve peak shapes. The elution gradient (*v*/*v*) was 100% B at 0 min, 60% B at 5 min, 30% B at 5.5 min, 30% B at 7 min, 15% B at 8.5 min, 100% B at 9 min, and 100% B at 22 min. The column flow rate was 0.3 mL/min; the column temperature was 35 °C, and the autosampler was kept at 10 °C. Data acquisition and LC-MS control were carried out using the Agilent MassHunter Data Acquisition software (version B06.00 Build 6.0.6025.4). The autosampler was kept at 10 °C. The QQQ-MS was operated in SRM mode in positive ion polarity using the following settings: capillary voltage, 4,000 V; drying N2 gas and temperature, 11 L/min and 125 °C respectively; and nebulizer, 15 psi. All optimized SRM transitions for each target are listed in Supplementary Table S3. Data analysis was carried out using MassHunter Quantitative Analysis Software (version 10.1, Build 10.1.733.0). Metabolites were quantified by comparing the integrated peak area with a calibration curve obtained using authentic standards, and normalized against fresh weight and internal standards.

### Statistical analysis

All statistical analyses were performed with Excel (Student's *t*-test) or RStudio (including analysis of variance with Tukey post hoc analysis, Shapiro–Wilk normality test, and Levene's Test). Statistical tests and replicate numbers are as indicated in figure legends. All statistical evidence is provided in Supplementary Data Set 3.

### Accession numbers

Sequence data from this article can be found GenBank/EMBL databases under the following accession numbers: ENOD93 (AT5G25940), ATHIGD1 (AT3G48030.1), ATHIGD2 (At5g27760) and ATHIGD3 (At3g05550), Actin7 (AT5G09810), RCF1 (YML030W), RCF2 (YNR018W) and RCF3 (YBR255C-A). All data generated or analyzed during this study are included in this published article and its supplementary information files. The raw mass spectrometry data are submitted to PRIDE, project accession PXD041903, for the LC-MS/MS protein identifications and project accession PXD041995 for the LC–SRM–MS peptide quantifications.

## Supplementary Material

koae242_Supplementary_Data

## Supplementary data

The following materials are available in the online version of this article.

**Supplementary Figure S1.** Enod93 and Rcf2c homologs are widespread across plants.

**Supplementary Figure S2.** Phylogenetically broad protein alignments demonstrate homology between ENOD93 and Hig1-domain proteins with the N- and C-terminus of Rcf2, respectively.

**Supplementary Figures S3.** Co-expression and intra-mitochondrial localization of ENOD93.

**Supplementary Figure S4.** Phenotype of *enod93* and a complemented line and identification of ENOD93.

**Supplementary Figure S5.** Succinate- or malate/glutamate-dependent respiration by isolated *enod93* mitochondria.

**Supplementary Figure S6.** Spin recovery assays of respiratory rate in isolated *enod93* mitochondria.

**Supplementary Figure S7.** Effect of lowering membrane potential and complex IV dependence based on differences between Col-0, enod93, and complemented line mitochondrial bioenergetics.

**Supplementary Figure S8.** Complex distribution of plant- and fungal-type Rcf2 homologs across the breadth of eukaryotes.

**Supplementary Table S1.** Identification of ENOD93 from 2D-BN/SDS–PAGE.

**Supplementary Table S2.** Primer sequences used in the study.

**Supplementary Table S3.** MRM parameters for detecting nucleotides.

**Supplementary Data Set 1.** Mitochondrial protein abundances determined by multiple reaction monitoring LC-MRM-MS from Col-0, *enod93*, and complemented line.

**Supplementary Data Set 2.** Metabolite analysis of roots and leaves of Col-0, *enod93*, and complemented lines.

**Supplementary Data Set 3.** All statistical analysis.

## Dive Curated Terms

The following phenotypic, genotypic, and functional terms are of significance to the work described in this paper:

ATP CHEBI: CHEBI:15422

ADP CHEBI: CHEBI:16761

NADH CHEBI: CHEBI:16908
