# Supplementary material for: EARLY NODULIN93 acts via cytochrome c oxidase to alter respiratory ATP production and root growth in plants
Source: Plant Cell. 2024 Aug 23;36(11):4716–31. doi: 10.1093/plcell/koae242 (PMC11530774; doi:10.1093/plcell/koae242)
Supplement: koae242_Supplementary_Data [file koae242_supplementary_data.zip › tpc.01142.2023-s09.pdf]

THE PLANT CELL

ENOD93 acts via cytochrome c oxidase to alter respiratory ATP production and root growth in plants

Chun-Pong Lee, Xuyen Le, Ryan Gawryluk, José Casaretto, Steven Rothstein, and A. Harvey Millar

Review Timeline:

Submission Date:	01-Dec-2023
Editorial Decision:	05-Jan-2024
Revision Received:	05-Jun-2024
Editorial Decision:	26-Jun-2024
Revision Received:	24-Jul-2024
Accepted:	25-Jul-2024

1st Editorial Decision 05-Jan-2024

Prof. A. Harvey Millar
The University of Western Australia
Perth, WA 6009
Australia

Dear Harvey:

We have received reviews of your manuscript entitled "ENOD93 interacts with cytochrome c oxidase altering respiratory ATP production and root growth in plants." Thank you for submitting your best work to The Plant Cell. The editorial board agrees that the work you describe is substantive, falls within the scope of the journal, and may become acceptable for publication, pending revision and potential re-review.

We ask you to pay attention to the following points in preparing your revision:

Below are the comments from two expert reviewers, both of whom support communication of this work, but recommend a number of revisions. Please address the reviewers' queries and suggestions to the best of your ability. We ask you to pay particular attention to the following two major points: Reviewers and Editors consider it particularly important that, in your revised manuscript, you provide more direct experimental evidence for ENOD93 interacting with the cytochrome c oxidase. All your current data would also be compatible with a scenario, in which the ATP synthase activity is impaired. Perhaps this point could be (at least indirectly) addressed by further characterizing how complex IV activity influences the ATP synthase activity, and how the membrane potential is affected in the enod93 mutants?

In addition, a better characterization of the overexpression lines will be required, to better support the proposed link between ENOD93 and nitrogen use efficiency, which was judged to be very preliminary at this stage by reviewers and editors.

Please contact us if there are ambiguous comments or if you wish to discuss the revision.

Given the nature of the comments, we are offering you 60 days from when we have issued this decision to complete the revision. If a revision is not returned within this time frame, and if you have not been granted an extension, we will withdraw the manuscript, which will leave you free to submit the work elsewhere. If you need an extension, we encourage you to contact us at any point before the 60 days have passed.

When you are ready to submit the revised version, please upload a highlighted copy that indicates all changes made in response to the editor and reviewer recommendations. Include an itemized list of all changes made in response to each of the reviewer's suggestions in the "Response to Reviewers" section; please note that reviewers do not have access to your cover letter, nor was this decision letter shared with them.

Thank you for the privilege of reviewing your work. We look forward to receiving your revised manuscript.

Sincerely,

The Plant Cell Editorial Board

Please note the following:

-The Plant Cell now requires authors to complete and submit an author revisions checklist upon submission of a revised manuscript. The aim of the checklist is to aid authors in preparing a high-quality manuscript, facilitate the review and assessment of revised manuscripts, and help to ensure that journal standards are maintained across the board. If your manuscript is accepted, the completed checklist will be published as supplemental material attached to the article online. Please download a copy of the checklist (pdf fillable form) at this link, for submission with your revised manuscript: https://tpc.msubmit.net/html/Author_Revisions_Checklist.pdf.

-Supplemental materials should be restricted to large datasets and tables, presentation of replicates, and validation of reagents, methods, or genotypes. Any data that are used to support the major claims must be in the main manuscript. Supplemental figure legends must indicate what figure in the main manuscript is supported by the supplemental data presented. Please justify how each of the supplemental figures meet the criteria.

-Sampling methods and nature of "biological replicates" should be described precisely (i.e. different plants, parts of

plants, pooled tissue, independent pools of tissue, sampled at different times, etc), along with a clear description of and rationale for any statistical analyses conducted. The reader should know exactly what was sampled; what forms the basis of the calculation of any means and statistical parameters reported. This is also necessary to ensure that proper statistical analysis was conducted.

-Want to add this revision deadline to your calendar? Click below!

--- Reviewer comments:

Reviewer #1 (Comments for the Author):

In their manuscript titled 'ENOD93 interacts with cytochrome c oxidase altering respiratory ATP production and root growth in plants' Lee et al. identify and characterize a so far unknown plant homolog of the yeast RCF2 subunit.

At the center of this study is the early nodulation 93 (ENOD93) protein, which has a proclaimed role in the formation of root nodules in legumes and supports nitrogen uptake in non-legumes. ENOD93 resembles the N-terminal domain of the yeast RESIPRATORY SUPERCOMPLEX 2 (RCF2) protein, while another Arabidopsis protein group containing HIGD2 and HIGD3 resemble the C-terminal part of RCF2. It is proposed that ENOD93 and the HIG proteins replace RCF2 in plants. This notion is supported by the finding that yeast RCF2 is post-translationally cleaved into two parts, resembling ENOD93 and HIG.

Importance of findings
ENOD93 has been identified already decades ago as potential complex IV subunit in plants, but no function could be associated with this protein. Lee et al. now demonstrate that the protein is not an essential complex IV subunit, but is associated with the complex. While it does not affect complex IV activity in vitro, its absence has a detrimental effect on in vivo complex IV performance. The fact that the activity of the complex IV depends on the membrane potential makes this mutant extremely interesting. Its characterization promises to produce insights into plant respiration at a level, which cannot be achieved by the analysis of central respiratory complex subunits. I therefore expect that the work on the enod93 mutant shown here is just the beginning.

Quality of experiments
Data provided by the authors is of high quality. Experiments were performed with great care and fully comply with scientific standards.

Points in favor
Despite my comments in the following paragraph (see below), the strong part of this manuscript is the physiological characterization of the enod93 knock-out mutant. The authors went to great length in order to describe how mutant respiration differs from its WT counterpart. The finding that complex IV performance in the enod93 mutant depends on membrane potential is genuinely interesting and is convincingly documented by the experiments carried out by the author team.

Points detracting
I wonder about the connection of membrane potential and altered complex IV capacity in the mutant. If complex IV activity is reduced, membrane potential would rise to a certain point due to the temporary continued activity of complexes I and III until ubiquinone and cytochrome c pools are saturated. This should also promote the formation of ROS. Did the authors notice anything in this direction? It is also not clear to me, why membrane potential increases over time, since a functional ATP-synthase should prevent this.
The authors claim in line 196 that "we identified ENOD93 in high molecular mass protein complexes above 650 kDa". I was struggling to find these data in the manuscript, but they could hint towards an interaction of ENOD93 with the ATP synthase complex usually migrating at 650-700 kDa in BN-PAGE.

Author actions necessary for acceptance
As pointed out above, the findings related to the knock-out of ENOD93 by the author team are highly interesting and rely on extensive and solid data. However, the burning question as to how the observed decrease of (in vivo) CIV activity and the increased membrane potential are interlinked remains unclear. It is understood that the work presented here can only be a starting point, but I still suggest that the authors discuss these points in more detail and, ideally, produce a hypothesis on how complex IV activity interlinks with the observed increased membrane potential in the enod93 mutant. It is know from research on mammalian mitochondria, that the proton gradient across the inner mitochondrial membrane is not homogenous, but that a lateral gradient between complex IV and the ATP-synthase complex exists (Rieger et al. 2014, Nat. Commun. 5: 3103), which could be disturbed in the ENOD93 mutant, and which may explain the observations described here.

Reviewer #2 (Comments for the Author):

The manuscript from Lee et al. describes the characterization of the ENOD93 protein in Arabidopsis. ENOD93 is a mitochondrial protein that is homologous to the N-terminal domain of the yeast RCF2 protein that is involved in the regulation of the cytochrome c oxidase of the oxidative phosphorylation system. The authors obtained a knock-out mutant and characterized the respiratory chain and the respiration of the enod93 mutant. They observed no alteration of the OXHPOS system but oxygen consumption and membrane potential were impaired. Altogether these results suggest that ENOD93 plays a role in the regulation of the OXPHOS system. This finding is very exciting as little is known about the regulation of respiratory fluxes in plants.

Proteins of the ENOD93 family were previously shown to be involved in nitrogen use efficiency in legumes and mutants lacking ENOD93 in these plants show altered amino acid accumulation profiles. The authors created lines overexpressing ENOD93 in Arabidopsis and they observed changes in the accumulation of several amino acid and metabolites in the mutants and overexpression lines. Altogether they propose that ENOD93 is involved in nitrogen use efficiency via its role in regulating mitochondrial ATP production.

This manuscript is clearly written and the data are of high quality. In general, all the controls and replicates experiments have been performed. However, there are a few points that could be improved to strengthen the conclusions of this study.

In the first part, the authors argue that ENOD93 interacts with the cytochrome c oxidase. They provide a list of indirect clues supporting they claim. However, they do not provide any experimental evidence that ENOD93 interacts with the cytochrome c oxidase. The comigration on native gel is not sufficient to conclude that proteins are present in the same complex. Also, in the MS analysis of the gel spot shown in Figure S4 and Table S1, ENOD93 is found in the same spot than an ATP synthase subunit (AT4G30010) which could suggest that ENOD93 rather interacts with the ATP synthase than with the cytochrome c oxidase. Identifying the place where ENOD93 interacts with the OXPHOS system would greatly improve this study.

In the second part, the authors produced overexpressor lines but they do not show any characterization of these lines. They write that in these lines, the expression of ENOD93 is 60 to 100 times higher than in the WT but the data are not presented. Considering that in the complemented lines, the ENOD93 transcript is about 4 times more abundant (Figure 2B) but the protein levels are not impacted (Figure 2D), quantification of the protein in the overexpression line is required to qualify these lines are overexpression. Also, the authors discuss that in the overexpression line, ENOD93 might enable higher ATP production to sustain N fixation but ATP levels measured in the overexpression lines are lower (Figure 7B). This part appears a bit preliminary and a deeper characterization of the overexpression lines would be required to better support the hypothesis proposed by the authors. Characterizing the respiration, in particular measuring the ATP production (as done in Figure 5A) of the overexpression line could be performed to back up the authors' claim.

Minor comments:
- Line 118 and 143, HMM should be explained.
- Figure 1 requires more explanation. In A, the red box is not explained, In C explain why the TM are in different colours. Also explain what is shown in the figure, why are there 6 rows with letters, why are the two panels identical, what is the meaning of the numbers and the arrowhead.
- Line 161, confusion gene/protein, the nine genes are nuclear encoded but the gene products are located in the mitochondria
- Lines 193-194-196, There is no signal at 600 kDa in the complexome shown in Figure S3. No sizes are indicated in Figure S4B. No data about the presence of ENOD93 in the 650 kDa complex is shown. Maybe it would be good to indicate on the figure all the bands that have been analysed and to provide all these data in the Table S1.
- Line 304, no data showing an interaction between ENOD93 and complex IV are shown, please modulate the statement
- Line 351, mitochondria instead of mitochondrial
- Line 536, K2H2PO4 is a chemical that does not exist
- Line 544, where the mitochondria broken before performing the assay with NADH?
- Line 568, in-gel staining of complex I is not shown in the manuscript

Project Name: ENOD93 interacts with cytochrome c oxidase altering respiratory ATP production and root growth in plants – blue native gel protein complex identification

Project accession: PXD041903

Reviewer account details:

Username: reviewer_pxd041903@ebi.ac.uk

Password: SfYS1wRH

Project Name: ENOD93 interacts with cytochrome c oxidase altering respiratory ATP production and root growth in plants – targeted analysis of peptides for mitochondrial proteins

Project accession: PXD041995

Username: reviewer_pxd041995@ebi.ac.uk

Password: Re7OasQn

Dear Editors

We have made revisions to our manuscript “ENOD93 interacts with cytochrome c oxidase altering respiratory ATP production and root growth in plants” with a slight change to the title to avoid any overemphasis “ENOD93 acts via cytochrome c oxidase to alter respiratory ATP production and root growth in plants”. We hope we have adequately addressed the comments of editor and reviewers and present a fair appraisal of the data on this challenging analysis. Our responses are in blue below. The first two authors are also now joint given the extra work conducted by Xuyen Le in this manuscript.

Regards

Harvey Millar

Dear Harvey:

We have received reviews of your manuscript entitled "ENOD93 interacts with cytochrome c oxidase altering respiratory ATP production and root growth in plants." Thank you for submitting your best work to The Plant Cell. The editorial board agrees that the work you describe is substantive, falls within the scope of the journal, and may become acceptable for publication, pending revision and potential re-review.

We ask you to pay attention to the following points in preparing your revision:

Below are the comments from two expert reviewers, both of whom support communication of this work, but recommend a number of revisions. Please address the reviewers' queries and suggestions to the best of your ability. We ask you to pay particular attention to the following two major points: Reviewers and Editors consider it particularly important that, in your revised manuscript, you provide more direct experimental evidence for ENOD93 interacting with the cytochrome c oxidase. All your current data would also be compatible with a scenario, in which the ATP synthase activity is impaired. Perhaps this point could be (at least indirectly) addressed by further characterizing how complex IV activity influences the ATP synthase activity, and how the membrane potential is affected in the enod93 mutants?

In addition, a better characterization of the overexpression lines will be required, to better support the proposed link between ENOD93 and nitrogen use efficiency, which was judged to be very preliminary at this stage by reviewers and editors.

Please contact us if there are ambiguous comments or if you wish to discuss the revision.

Given the nature of the comments, we are offering you 60 days from when we have issued this decision to complete the revision. If a revision is not returned within this time frame, and if you have not been granted an extension, we will withdraw the manuscript, which will leave you free to submit the work elsewhere. If you need an extension, we encourage you to contact us at any point before the 60 days have passed.

When you are ready to submit the revised version, please upload a highlighted copy that indicates all changes made in response to the editor and reviewer recommendations. Include an itemized list of all changes made in response to each of the reviewer's suggestions in the "Response to Reviewers" section; please note that reviewers do not have access to your cover letter, nor was this decision letter shared with them.

Thank you for the privilege of reviewing your work. We look forward to receiving your revised manuscript.

Sincerely,

The Plant Cell Board of Editors

Response to editors: There are three issues raised here that we will seek to respond to, and then directly address reviewer points one-by-one below.

1. **Does ENOD93 directly interact/bind to Complex IV?** The yeast literature on RCF2 has been controversial over more than a decade between its discovery, evidence of association with complex IV containing complexes on BN-PAGE, but absence from crystal structures. There is no evidence of RCF2 interaction with Complex IV in yeast in two hybrid studies to our knowledge, testifying to a weak interaction between small hydrophobic proteins that is hard to stabilise. Only a chance discovery in particular types of complex IV preparations for cryoEM defined its site of association in yeast in 2021 and 2023 (amongst over 40 PDB structures of complex IV without it). In comparison we think the BN-PAGE (and revised BN-PAGE complexome presentations in Supp Figure 3) are fair evidence that the same situation exists in plants. There is no evidence of HIGD2 or ENOD93 in the only plant Complex IV in the single CryoEM publication on plants (Maldonado et al 2021), but the authors did report that during purification HIGD2 was present but did not appear in averaged final structures. We have recently attempted binary yeast-two hybrid interaction assays in the past few months between Arabidopsis HIGD2, ENDO93 and COX6B using a research service on the off chance that this might work, but this has found no convincing evidence of interaction. While this is disappointing, we consider our current evidence is consistent with what is known in yeast and we cannot define a rapid way to address this issue further without an extended period of experimentation with no clarity of a positive result.

2. **Could our ENOD93 results arise from only a direct effect on Complex V rather than Complex IV.** There is ample evidence against this hypothesis.
   A) It is clear the BN-PAGE association is with complex IV and not with pattern of ATP synthase complexes (see revised supplemental figure 3)
   B) The membrane potential effect seen in ENOD93 is relieved by removing complex IV from the ETC using FeCN acceptance from cytochrome c, indicating it depends on complex IV and does not operate when complex IV is not engaged in the ETC.
   C) There is no known mechanism in any other organism for RCF2 like proteins to be acting directly on ATP synthase, but there is for complex IV.

3. **The data and interpretation of the overexpression phenotypes is preliminary and the link to NUE as shown in rice studies remains preliminary.** We tend to agree with this and despite the fact we have some data and they are intriguing and consistent with aspects of previous reports on ENOD93, in this revision we have removed OE lines from the MS and focus only on the mutant and complementation lines to document effect of ENOD93 loss. We retain some of the discussion on ENOD93 literature in other plants because we think it is really important to make this connection to stimulate research on this gene family. Based on requests from reviewers we also propose a tentative hypothesis to explain ENOD93 action in mitochondria and its probable secondary impact on mitochondrial plant ATP synthesis, metabolism and phenotype. This change has removed the need for several supplemental figures of metabolite levels. Instead we have modified Figure 8 just to give the

key metabolites that change in enod93, and all the remaining metabolite data from WT, *enod93* and complemented line are now provided in table format in Table S5.

We also slightly revise comments on the ENOD93 phenotype as we now have convincing evidence of early flowering linked to ENOD93 loss.

The Plant Cell

Please note the following:</>

-The Plant Cell now requires authors to complete and submit an author revisions checklist upon submission of a revised manuscript. The aim of the checklist is to aid authors in preparing a high-quality manuscript, facilitate the review and assessment of revised manuscripts, and help to ensure that journal standards are maintained across the board. If your manuscript is accepted, the completed checklist will be published as supplemental material attached to the article online. Please download a copy of the checklist (pdf fillable form) at this link, for submission with your revised manuscript: https://tpc.msubmit.net/html/Author_Revisions_Checklist.pdf.

-Supplemental materials should be restricted to large datasets and tables, presentation of replicates, and validation of reagents, methods, or genotypes. Any data that are used to support the major claims must be in the main manuscript. Supplemental figure legends must indicate what figure in the main manuscript is supported by the supplemental data presented. Please justify how each of the supplemental figures meet the criteria.

-Sampling methods and nature of "biological replicates" should be described precisely (i.e. different plants, parts of plants, pooled tissue, independent pools of tissue, sampled at different times, etc), along with a clear description of and rationale for any statistical analyses conducted. The reader should know exactly what was sampled; what forms the basis of the calculation of any means and statistical parameters reported. This is also necessary to ensure that proper statistical analysis was conducted.

-Want to add this revision deadline to your calendar? Click below!

-- Reviewer comments:

Reviewer #1 (Comments for the Author):

In their manuscript titled 'ENOD93 interacts with cytochrome c oxidase altering respiratory ATP production and root growth in plants' Lee et al. identify and characterize a so far unknown plant homolog of the yeast RCF2 subunit.

At the center of this study is the early nodulation 93 (ENOD93) protein, which has a proclaimed role in the formation of root nodules in legumes and supports nitrogen uptake in non-legumes. ENOD93 resembles the N-terminal domain of the yeast RESIPRATORY SUPERCOMPLEX 2 (RCF2) protein, while another Arabidopsis protein group containing HIGD2 and HIGD3 resemble the C-terminal part of RCF2. It is proposed that ENOD93 and the HIG proteins replace RCF2 in plants. This notion is supported by the finding that yeast RCF2 is post-translationally cleaved into two parts, resembling ENOD93 and HIG.

Importance of findings
ENOD93 has been identified already decades ago as potential complex IV subunit in plants, but no function could be associated with this protein. Lee et al. now demonstrate that the protein is not an essential complex IV subunit, but is associated with the complex. While it does not affect complex IV activity in vitro, its absence has a detrimental effect on in vivo complex IV performance. The fact that the activity of the complex IV depends on the membrane potential makes this mutant extremely interesting. Its characterization promises to produce insights into plant respiration at a level, which cannot be achieved by the analysis of central respiratory complex subunits. I therefore expect that the work on the enod93 mutant shown here is just the beginning.

Quality of experiments
Data provided by the authors is of high quality. Experiments were performed with great care and fully comply with scientific standards.

Points in favor
Despite my comments in the following paragraph (see below), the strong part of this manuscript is the physiological characterization of the enod93 knock-out mutant. The authors went to great length in order to describe how mutant respiration differs from its WT counterpart. The finding that complex IV performance in the enod93 mutant depends on membrane potential is genuinely interesting and is convincingly documented by the experiments carried out by the author team.

Points detracting
I wonder about the connection of membrane potential and altered complex IV capacity in the mutant. If complex IV activity is reduced, membrane potential would rise to a certain point due to the temporary continued activity of complexes I and III until ubiquinone and cytochrome c pools are saturated. This should also promote the formation of ROS. Did the authors notice anything in this direction? It is also not clear to me, why membrane potential increases over time, since a functional ATP-synthase should prevent this.

Response: We are not convinced we see progressive membrane potential rising over time, but instead we see PMF maintained at a higher level in *enod93* mitochondria from the very beginning of experiments. We conclude that in the absence of ENOD93 the PMF is allowed or enabled to rise to a higher level than is normal in WT and this may directly or indirectly contribute to the time-dependent loss of complex IV-dependent oxygen consumption. Higher PMF in the mutant might be a consequence or a response to ENOD93 loss. The higher sensitivity of the mutant root phenotype to uncouplers and faster respiration on a tissue mass basis may indicate it is a response and higher PMF helps sustain mitochondrial function in the absence of this protein *in vivo*. We do not see any rise in the abundance of the classical AOX, NDHs or UCP in *enod93* plant mitochondria (Supplemental data on MRMs Supp Table S2) nor evidence of more KCN-insensitive respiratory capacity in respiratory assays (see Figure 2, Figure S5) in *enod93*, so we have no reason to think there is a ROS-linked effect here.

The authors claim in line 196 that "we identified ENOD93 in high molecular mass protein complexes above 650 kDa". I was struggling to find these data in the manuscript, but they could hint towards an interaction of ENOD93 with the ATP synthase complex usually migrating at 650-700 kDa in BN-PAGE.

Response: This comment is based on low level evidence of ENOD93 from BN-PAGE gels from complexome maps (Figure S3). Indeed in yeast RCF2 is named as such – being a factor associated with different supercomplexes - but ultimately the site of attachment has been shown to be complex IV in yeast and evidenced as such also in CryoEM structures. We also have gone down the rabbit hole of looking for a direct ATP synthase link in our exploration of the phenomena we observe, but the weight of the evidence is against it. We now more directly deal with ATP synthase hypothesis in the manuscript in several ways.

1. We note that our detailed assessment of Complex V protein abundances (Table S2), BN-PAGE abundance (Figure 4,) show no difference in *enod93*.
2. We provide direct comparison of ATP synthase subunit location vs Complex IV and ENOD93 in complexome maps showing they are distinct in a revised Figure S3.

Author actions necessary for acceptance
As pointed out above, the findings related to the knock-out of ENOD93 by the author team are highly interesting and rely on extensive and solid data. However, the burning question as to how the observed decrease of (in vivo) CIV activity and the increased membrane potential are interlinked remains unclear. It is understood that the work presented here can only be a starting point, but I still suggest that the authors discuss these points in more detail and, ideally, produce a hypothesis on how complex IV activity interlinks with the observed increased membrane potential in the enod93 mutant. It is know from research on mammalian mitochondria, that the proton gradient across the inner mitochondrial membrane is not homogenous, but that a lateral gradient between complex IV and the ATP-synthase complex exists (Rieger et al. 2014, Nat. Commun. 5: 3103), which could be disturbed in the ENOD93 mutant, and which may explain the observations described here.

Response: We appreciate the reviewer's comments and recognise that our range of observations are complex to reconcile in a simple model. These experiments were in fact performed when we were probing both ideas of a Complex IV or a Complex V action. We previously wanted to just provide the evidence of the importance of ENOD93 rather than potentially overinterprete experiments to propose a mechanism that is still not definitive. However, we see the value of clarity for readers so we have made a hypothesis based on the evidence in a revised section of the discussion, based on what is known in yeast and mammalian mitochondrial bioenergetics and what we observe.

Our now state that ENOD93 appears to have an initiating site of action at complex IV. This is evidenced by BN-PAGE association, progressive loss of respiration being dependent on complex IV (FeCN) and independent of external ND, complex I, complex II delivery of electrons (i.e. same progressive losses using external NADH, Succ or TCA cycle substrates Supp Figure S5), and lack of effects during complex I to complex III electron transport and proton translocation (FeCN experiment). But coupled to this primary effect is also a secondary consequence of *enod93* loss on ATP synthesis and potential Complex IV: ATP synthase dependences. This is evidenced by slowed respiration rate via complex IV and ATP synthesis rate even under PMF, plus the reversibility of the progressive inhibition by uncouplers and membrane deenergisation. Supercomplex kinetics and in organelle interactions that remain to be determined in plants and/or non-homogenous mitochondrial PMF and a lateral PMF gradient between complex IV and the ATP-synthase complex observed in mammals and yeast could be contributors.

Reviewer #2 (Comments for the Author):

The manuscript from Lee et al. describes the characterization of the ENOD93 protein in

Arabidopsis. ENOD93 is a mitochondrial protein that is homologous to the N-terminal domain of the yeast RCF2 protein that is involved in the regulation of the cytochrome c oxidase of the oxidative phosphorylation system. The authors obtained a knock-out mutant and characterized the respiratory chain and the respiration of the enod93 mutant. They observed no alteration of the OXHPOS system but oxygen consumption and membrane potential were impaired. Altogether these results suggest that ENOD93 plays a role in the regulation of the OXPHOS system. This finding is very exciting as little is known about the regulation of respiratory fluxes in plants.

Proteins of the ENOD93 family were previously shown to be involved in nitrogen use efficiency in legumes and mutants lacking ENOD93 in these plants show altered amino acid accumulation profiles. The authors created lines overexpressing ENOD93 in Arabidopsis and they observed changes in the accumulation of several amino acid and metabolites in the mutants and overexpression lines. Altogether they propose that ENOD93 is involved in nitrogen use efficiency via its role in regulating mitochondrial ATP production.

This manuscript is clearly written and the data are of high quality. In general, all the controls and replicates experiments have been performed. However, there are a few points that could be improved to strengthen the conclusions of this study.

In the first part, the authors argue that ENOD93 interacts with the cytochrome c oxidase. They provide a list of indirect clues supporting they claim. However, they do not provide any experimental evidence that ENOD93 interacts with the cytochrome c oxidase. The comigration on native gel is not sufficient to conclude that proteins are present in the same complex. Also, in the MS analysis of the gel spot shown in Figure S4 and Table S1, ENOD93 is found in the same spot than an ATP synthase subunit (AT4G30010) which could suggest that ENOD93 rather interacts with the ATP synthase than with the cytochrome c oxidase. Identifying the place where ENOD93 interacts with the OXPHOS system would greatly improve this study.

Response: We are also very interested in this idea of evidence for ENOD93 interaction with ATP synthase, we think our BN-PAGE spot analysis in Figure S4 is simply not accurate enough to conclude an interaction that would be contrary to what is known from extensive work in yeast and also the much higher resolution and systematic analysis of complexomes performed by the Braun group in Hannover (Supplemental figure 3) that clearly show the association to the two types of complex IV (Iva and IVb) and complex IV subcomplexes, and not to the patterns for ATP synthase and its subcomplexes. As has been found in yeast for RCF2, there is no evidence from large scale protein-protein interaction studies of ENOD93 interaction in Arabidopsis. In addition, we have performed a pair-wise yeast two hybrid assays to probe HIGD2, ENOD93, COX6b interactions with a research service that has been inconclusive. There is no plant complex IV crystal structure and only one plant Complex IV CryoEM structure that contains neither HIGD2 or ENOD93. It is evident that proving association of two small hydrophobic proteins with a membrane embedded complex that may include the surfaces of multiple hydrophobic proteins of that complex is challenging.

In the second part, the authors produced overexpressor lines but they do not show any characterization of these lines. They write that in these lines, the expression of ENOD93 is 60 to 100 times higher than in the WT but the data are not presented. Considering that in the complemented lines, the ENOD93 transcript is about 4 times more abundant (Figure 2B) but the protein levels are not impacted (Figure 2D), quantification of the protein in the overexpression line is required to qualify these lines are overexpression. Also, the authors

discuss that in the overexpression line, ENOD93 might enable higher ATP production to sustain N fixation but ATP levels measured in the overexpression lines are lower (Figure 7B). This part appears a bit preliminary and a deeper characterization of the overexpression lines would be required to better support the hypothesis proposed by the authors. Characterizing the respiration, in particular measuring the ATP production (as done in Figure 5A) of the overexpression line could be performed to back up the authors' claim.

Response: We acknowledge that the overexpressing lines and their characterisation is preliminary, and it remains ongoing, and it will need considerably more work to reach the level of conclusions possible for the mutant. To simplify the claims in the manuscript and avoid over interpretation we have now removed the OE lines from the manuscript and focus instead only on the mutant and its complementation to provide a single focus for the manuscript in elucidating the key features of ENOD93 action through its genetic disruption in Arabidopsis.

Minor comments:
- Line 118 and 143, HMM should be explained.

Expanded and cited

- Figure 1 requires more explanation. In A, the red box is not explained, In C explain why the TM are in different colours. Also explain what is shown in the figure, why are there 6 rows with letters, why are the two panels identical, what is the meaning of the numbers and the arrowhead

Expanded and explained.

- Line 161, confusion gene/protein, the nine genes are nuclear encoded but the gene products are located in the mitochondria

Text modified to avoid confusion

- Lines 193-194-196, There is no signal at 600 kDa in the complexome shown in Figure S3. No sizes are indicated in Figure S4B. No data about the presence of ENOD93 in the 650 kDa complex is shown. Maybe it would be good to indicate on the figure all the bands that have been analysed and to provide all these data in the Table S1.

The evidence of ENOD93 peptide signals above 600 kDa in Figure S3 were very faint, we have increased contrast to see them. We have added sizes to Figure S4B for easier comparison to Figure S3.

In response to requests about complex V we had modified Figure 3 to include ATP synthase subunits to show its distinct banding patterns.

Limited BN-PAGE gel spot IDs alone are not very helpful without being comprehensive and/or quantitative to prove 'here, but not here, claims' . Our analysis and conclusions are thus led by the complexome studies from re-analysis of Senkler et al 2017 data and we just sought to confirm

ENOD93 at the location claimed, show its absence in the mutant and its return in the complemented line.

- Line 304, no data showing an interaction between ENOD93 and complex IV are shown, please modulate the statement

We believe the BNPAGE data are important evidence in favour of interaction, but also that the claim in not necessary to make in that sentence without caveat, so have removed the statement.

- Line 351, mitochondria instead of mitochondrial - corrected
- Line 536, K2H2PO4 is a chemical that does not exist -corrected

- Line 544, where the mitochondria broken before performing the assay with NADH? –

no, it is ATP production in intact mitochondria with NADH acting through the external NADH dehydrogenase and the ADP provided must enter the mitochondria.

- Line 568, in-gel staining of complex I is not shown in the manuscript –

This has been corrected, only Complex IV is reported in Figure 4B.

Dear Harvey:

We have received reviews of your manuscript entitled "ENOD93 acts via cytochrome c oxidase to alter respiratory ATP production and root growth in plants." On the basis of the advice received, the board of reviewing editors would like to accept your manuscript for publication in The Plant Cell. This acceptance is contingent on revision based on the comments of our reviewers.

Please highlight all changes and include a detailed annotation of changes of the text, with line numbers, and noting your responses to the comments.

A note from the Editor-in-Chief: We are trying to make a concerted effort to change green/red comparisons to green/magenta or other colors to make our figures understandable to those with color vision deficiencies. We noticed that a most of the bar and line graphs in your figures utilize red/green contrasts, so as you prepare the final version of the figures, please check and see if you can swap out these colors to make them more accessible. Please note that color changes do not need to be highlighted or tracked in the revised manuscript, but could be noted in the cover letter or response document.

To submit your revised manuscript, click:

Link Not Available

If you have any questions about the revision submission procedures, please contact the Editorial Office Staff (tpc-submissions@aspb.org). If you cannot return the revised manuscript within 30 days, please let us know. Otherwise, we will assume that you have elected not to revise the manuscript and withdraw it.

Thank you very much for the privilege of reviewing this work. I look forward to receiving the next version.

Sincerely,

The Plant Cell Board of Editors

Reviewer #1 (Comments for the Author):

In this revised version of the manuscript, the authors have convincingly responded to my comments and critique. In particular,I appreciate their effort in clarifying the association of enod93 with OXPHOS components (i.e. with complex IV and not complex V).

Apart from the minor typos outlined below, I therefore fully support the manuscript in its current form and its publication in TPC.

Line 31: "We show profile hidden Markov models define ENOD93...". Something with this sentence seems to be missing/odd/wrong

Line 121: "...that include the Hig_1_N domain". Should that perhaps read "...do NOT include the Hig_1_N domain", as suggested in Fig. 1?

Line 186: Fig. 2B does not seem to show BN/SDS-gels.

Line 362: blank missing between "mitochondria" and "lacking"

Line 396: blank missing between "reported" and "links"

Reviewer #2 (Comments for the Author):

All the points I have raised have been addressed. I do not have additional comments.

Please also note the following:

-The Plant Cell now requires authors to complete and submit an author revisions checklist upon submission of a revised manuscript. The aim of the checklist is to aid authors in preparing a high-quality manuscript, facilitate the review and assessment of revised manuscripts, and help to ensure that journal standards are maintained across the board. If your manuscript is accepted, the completed checklist will be published as supplemental material attached to the article online. Please download a copy of the checklist (pdf fillable form) at this link, for submission with your revised manuscript: https://tpc.msubmit.net/html/Author_Revisions_Checklist.pdf.

-Supplemental materials should be restricted to large datasets and tables, presentation of replicates, and validation of reagents, methods, or genotypes. Any data that are used to support the major claims must be in the main manuscript. Supplemental figure legends must indicate what figure in the main manuscript is supported by the supplemental data presented. Please justify how each of the supplemental figures meet the criteria.

-Sampling methods and the nature of "biological replicates" should be described precisely (i.e. different plants, parts of plants, pooled tissue, independent pools of tissue, sampled at different times, etc.), along with a clear description of and rationale for any statistical analyses conducted. The reader should know exactly what was sampled; what forms the basis of the calculation of any means and statistical parameters reported. This is also necessary to ensure that proper statistical analysis was conducted.

24/07/2024

Dear Editors

We have made final changes to the accepted TPC2023-RA-01142R1 as requested. These include the changes of red to magenta in figures as requested by editor, and modifications according to the final requests by reviewers (noted below). We have also consolidated supplemental files as requested and referred to these consolidations in the text that has changes some references out to supplemental datasets.

Regards

Harvey Millar

Rev 1 requests:

Line 31: "We show profile hidden Markov models define ENOD93...". Something with this sentence seems to be missing/odd/wrong

Changed to "We show that hidden Markov model define"

Line 121: "...that include the Hig_1_N domain". Should that perhaps read "...do NOT include the Hig_1_N domain", as suggested in Fig. 1?

Actually, our text is correct, this sentence is referring to finding HIG like domain proteins in plants that don't contain the N-terminus of RCF2.

Line 186: Fig. 2B does not seem to show BN/SDS-gels.

Correct this should refer to Supp Figure S4D and has been changed

Line 362: blank missing between "mitochondria" and "lacking"
Corrected

Line 396: blank missing between "reported" and "links"

Corrected

Dear Harvey / Dr. Millar:

We are pleased to inform you that your paper entitled "ENOD93 acts via cytochrome c oxidase to alter respiratory ATP production and root growth in plants" has been accepted for publication in The Plant Cell, pending a final editorial review by a science editor. At this stage, your manuscript will be evaluated by a science editor with respect to its presentation of scientific content, compliance with journal policies, and presentation for a broad readership. The Plant Cell has contracted with Plant Editors (planteditors.com) to provide this service to our authors, and you will soon receive additional information on this process.

Please note that each author needs to link their ORCID identifier to their account in the system before your manuscript can be published. If any authors do not have an ORCID linked to their account, they will receive a message with a link to complete this task. Please ensure that ALL of your coauthors have completed this task as soon as possible.

ASPB offers an OPEN option that allows authors to have their online articles available for free to all users immediately upon publication. For more information about the ASPB OPEN option, refer to the Final Submission Checklist Form.

The Plant Cell and The Arabidopsis Information Resource (TAIR) are collaborating to collect functional annotation data about Arabidopsis genes from authors. This includes information about the gene's molecular function (e.g., kinase activity, ATP synthetase activity), the biological process/es it is involved in (e.g., endosperm development, threonine biosynthesis), its subcellular location (e.g., nucleus, ER), anatomical or developmental expression pattern (e.g., leaf, ovule, flower stage 10, seedling stage), or its partner in a protein-protein interaction (e.g., AT1G01010 interacts with AT1G01020).

If your paper contains results falling into one or more of these categories for Arabidopsis genes, we request that you now submit these data for inclusion in TAIR by filling in the form provided at the following URL: https://www.arabidopsis.org/doc/submit/functional_annotation/123. If you need further clarification on what types of data can be submitted please contact curator@arabidopsis.org.

Finally, we encourage your submission of artwork for the journal cover. Monthly issues will have an online cover image and selected covers will be used for posters, other promotional items, and "wallpaper" for mobile devices. For more information, refer to Cover Submission in the Instructions for Authors https://tpc.msubmit.net/cgi-bin/main.plex?form_type=display_auth_instructions.

We look forward to seeing your paper published.

Sincerely,

The Plant Cell Board of Editors ----------------

=====
IMPORTANT REMINDER: PEER REVIEW REPORTS
=====

If you opted to publish a peer review report along with your article during the original submission process, it will be prepared by the editorial staff and publicly posted with your manuscript, inside the zip file that contains any other supplemental material. As a reminder, the peer review report is a public record of all comments from editors and reviewers, as well as your prior responses, as you received them in the decision letters for each draft of your manuscript. If you agreed to publish this report and have changed your mind, or are not sure if you selected this option, please contact the editorial office as soon as possible before signing the license agreement from our publisher.

=====

----FOR ASPB OFFICE USE ONLY (DO NOT EDIT)----
MSID: 37212
Corresponding Author: Prof. Millar
