# Supplementary material for: EARLY NODULIN93 acts via cytochrome c oxidase to alter respiratory ATP production and root growth in plants
Source: Plant Cell. 2024 Aug 23;36(11):4716–31. doi: 10.1093/plcell/koae242 (PMC11530774; doi:10.1093/plcell/koae242)
Supplement: koae242_Supplementary_Data [file koae242_supplementary_data.zip › TPC2023RA01142R2_Supplemental_Fig1_8_Tables1_3.pdf]

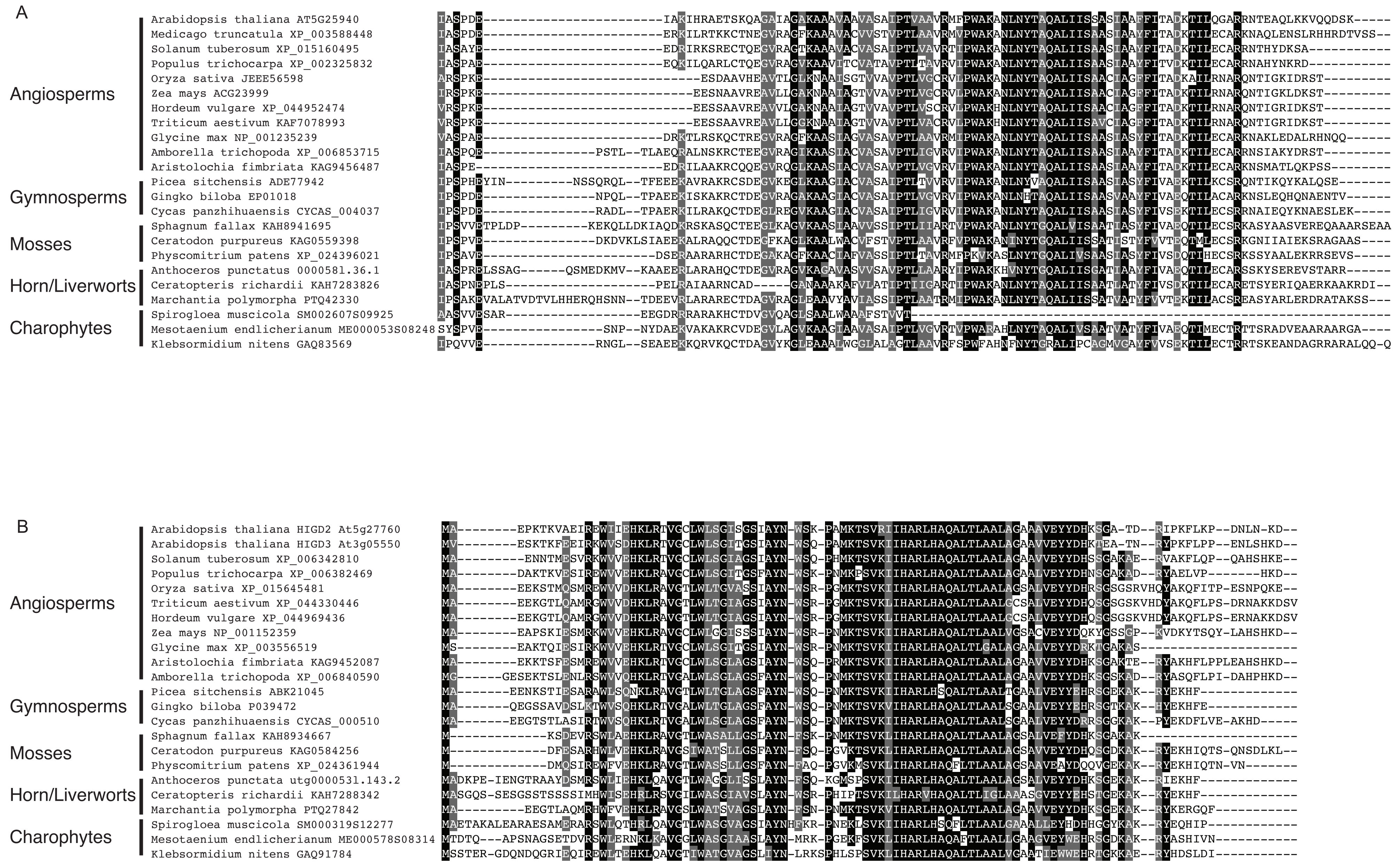

Figure S1 ENDO93 and Rcf2 homologs are widespread across plants. A) Multiple alignment of ENOD93 homologs from diverse angiosperms, gymnosperms, mosses, hornworts, liverworts, and charophyte algae. A small and poorly aligned N-terminal portion was removed for presentation. B) Multiple alignment of Rcf2 homologs from diverse angiosperms, gymnosperms, mosses, hornworts, liverworts, and charophyte algae. Shaded characters indicate positions with ≥80% sequence identity (black) or similarity (gray), defined according to amino acids belonging to groups with similar physicochemical properties (GAVLI, FYW, CM, ST, KRH, DENQ, P).

A

ENOD93 vs Rcf2 N-terminus

```
                                                                        TM helix 1                                          TM helix 2
(E) Arabidopsis thaliana AT5G25940             AKIHRAETSKQAGAIAGA-----------KAAVAAVASAIPTVAAVRM-FPWAKAN----LNYTAQALIISSASIAAFFITADKTILQ------------ 97
(E) Physcomitrium patens XP_024396021          ERAARARHCTDEGAKAGF-----------KACIAFVASSIPTLTAVRM-FPKVKAS----LNYTGQALIVSAASIASYFIVSDQTIHE------------ 112
(E) Marchantia polymorpha PTQ42330             VRLARARECTDAGVRAGL-----------EAVYAVIASSIPTLAATRM-IPWAKAN----LNYTAQALIISSATVATYFVVTEKTILA------------ 135
(E) Klebsormidium nitens GAQ83569              EKKQRVKQCTDAGVYKGL-----------EAALWGGLALAGTLAAVRF-SPWFAHN----FNYTGRALIPCAGMVGAYFVVSEKTILE------------ 117
(E) Cyanophora paradoxa g4415.t1              YGSGRFAVCADEVILESA-----------KGFGLAFTFAGVCERLFGKK-FPTFAR-----LSPTLRFYSVLAVGMVGFSYWGETAMQE------------ 103
(E) Hemiselmis andersenii CAMPEP_0172057764   DTVEQLNKIRQDRFNNGVMEA-C------KAAIWGTLSGLVLFGLRSVEEKTFRSTALARMGTPRLVYLTTGMACIGFALRGEQQLTQ------------ 173
(E) Acanthamoeba castellanii XP_004353487      PSEAQMESCRQQFLQEGG-----------KALAKWGGAATLFT-SAMSL-WPAYRNL----FNWRGQIFCVMGAGIAGFWIRGEQAVLR------------ 78
(E) Dictyostelium discoideum XP_643211         RRHKEEDETRSIFYKEGL-----------KGGLIFSGVATTGIAAACFK-SHTFKKS----VTWNIRAFLVSSAFIAGFWIYGETASMK------------ 110
(E) Diphylleia rotans SRT_a16321;84.p2         ESRRRMEECRGVFLREGV-----------KGAAIYSVITAAAVFAAHRS-WPLFQK-----QTLAFKVFIVSGGATCGMMIEGENAAHD------------ 92
(E) Thecamonas trahens XP_013760312            -------------LVNGDINAYCWSKLYPAWGVGMAVAGIPALAFVTL-APIAKR-----MPISARSGIAVSALVAGGWVNSETTVMS------------ 75
(R) Saccharomyces cerevisiae NP_014415         LTQDEIEAHRSHTLKGGI-----------EGALAGFAISAIIFKVLPRR-YPKFKPST---LTWSIKTALWITPPTVLTAICAEEASNN---FDATMYGSG 86
(R) Mantamonas plastica Gene24480              EERRLIEHTAGINMIEGLI----------KG----IVPAGILTLLANKY-WPAFRS-----WGTSPKMIIVYGAGIAAGYIQAEEAVMG------------ 122
(R) Capsaspora owczarzaki XP_004364750         ITQSELRNMRSELVRAGV-----------VGSAWTVAATSGAIFALSRL-SPAIAR-----TTIPSRVFVVTMLSTGVGVSCAERRMLAFRSESRAAKAAA 105
(R) Phytophthora infestans KAF4144666          NPNKRRDAIAWHAYTSGV-----------KTGAMTASVVGAGVLSANKY-WPAFRNR----LNTSGKTALVVMSFLASFTVEAENRLVH-GAHNPDIYMAT 97
(R) Ectocarpus siliculosus 299472452           EKRGRVLRLAQE---EGM-----------KAGGLAGLLSCAGVVAANKF-SPWFRKS----TSVSAKTALAVSPFFLFYLLRTELVMHG-ANHDPHAYGIV 91
```

B

Rcf2-c vs Rcf2 C-terminus

```
                                                              TM helix 3                                  TM helix 4
(C) Arabidopsis thaliana At5g27760            --------KTKVAEIREWIIEHKLRTVGCLWLSGISGSIAYNWSK-PAMKTSVRIIHARLHAQALTLAALAGAAAVEYYDHKSGAT-------------D 78
(C) Physcomitrium patens XP_024361944         ----------DMQSIREWFVEHKLRAVGTLWASSLLGSFAYNFAQ-PGVKMSVKLIHARLHAQFLTLAALAGSAAVEAYDQQVGEK-------------A 76
(C) Marchantia polymorpha PTQ27843            --------EGTLAQMRHWFVEHKLRAVGSLWATSVAGSLAYNFSN-PNMKTSVKVIHARLHAQALTLAALAGAAVVEYYDHKSGEK-------------A 78
(C) Klebsormidium nitens GAQ91784             --------QGRIEQIREWLTEHKLQAVGTIWATGVAGSLIYNLRKSPHLSPSVKLIHARLHAQALTLAALVGAATIEWWEHRTGKK-------------A 85
(C) Diphylleia rotans 178.p1                  --------YKQHQAKDGNMDVSPFKAAVSLWSAAIVGSLAYNWKN-PHLTQSQKMIHTRIYAQAITLAAVVGFGIYSSLHEEEKPK-------------- 77
(C) Thecamonas trahens XP_013755253           --------REDTLDNKTTPFWTPLKTVGALWLATLGGTLAYQFRN-NSLKLSQRVVQARVGAQAVTLGALIASAVVTVATESAAAS-------------P 78
(R) Saccharomyces cerevisiae NP_01441        KS------LSTKDKFVEGLSNNKYKIITGAWAASLYGSWVIVNKD-PIMTKAQKIVQARMYAQFITVGLLLASVGLSMYENKLHPN-------KQKVNEM 172
(R) Ustilago maydis 521 46099841             RTQSEWNSLSTRDKALTWAKENKFSVVAATWASSMVGIFAYIHTL-P-MSFAQKLVQARVWAQGITLASLVGMAAITQIPSKGDSI-I---RQHKEAADH 176
(R) Mantamonas plastica Gene24480            EKF-----AKKEEETIGLIVENRYSIVGGVWVASMIGSIAAWGN-PNLRTSQKVIHARLYAQAATLAALALGGVAASLSDTKHQN------PFEEIN-- 159
(R) Capsaspora owczarzaki XP_004364750       ANMSTW-EAVRTMDWNRWLIVNKFYVIGGIWAASMVGSLALSFRN-PNIKPSQKLIHARVYAQGLTVAAVVASAAVAPFDHKGDQE-------EKDAMDL 186
(R) Phytophthora infestans KAF4144666        K-------LKLWQRGANFLYDHPYRALASVGVPLVGGIYSYQHLN-KGISASQQVMHTRIYGQAAVVVLLLSSMAFHDYIAKRGKF-----GEEEEAH-- 173
(R) Ectocarpus siliculosus 299472452         KILQPRKELNAAQQLANWCYVNPFKMVVGMGVPAVGGILYSMRNE-GNLKLSQKLIHTRVYGQMTVVSMAVAMMAFQETMRRRGGLFVVTEAEEEEEEEE 187
```

Figure S2. Phylogenetically broad protein alignments demonstrate homology between ENOD93 and Hig1-domain proteins with the N- and C-terminus of Rcf2, respectively. A) Partial multiple alignment of putative ENOD93 homologs with the N-terminal region of putative Rcf2 from diverse eukaryotes. B) Partial multiple alignment of Hig1-domain proteins with the C-terminal region of putative Rcf2 homologs from diverse eukaryotes. Transmembrane helices of yeast Rcf2 are marked with solid lines according to Zhou et al. (2021). Shaded characters indicate positions with ≥80% sequence identity (black) or similarity (gray), defined according to amino acids belonging to groups with similar physicochemical properties (GAVLI, FYW, CM, ST, KRH, DENQ, P). Dark circles with an 'E' indicated putative Enod93 homologs; dark circles with an 'H' indicated Hig1-domain proteins equivalent to the C-terminal region of Rcf2; and white circles with an 'R' indicate full-length Rcf2 homologs.

A

No.	ATTED II Co-expression average	AGI number	Description	Subcellular location prediction SUBACON	Mitochondrial Complexome Map
1	8.28	AT4G16380	Heavy metal transport/detoxification protein	nucleus	no
2	7.95	AT2G40765	transmembrane protein	mitochondrion	YES
3	6.79	AT2G20820	hypothetical protein	mitochondrion	no
4	6.49	AT2G42680	multiprotein bridging factor 1A	nucleus	no
5	6.47	AT5G08060	unknown function protein	mitochondrion	YES
6	5.99	AT5G27760	Hypoxia-responsive family protein (HIG2)	mitochondrion	YES
7	5.85	AT1G80230	Cytochrome c oxidase, subunit Vb family protein	mitochondrion	YES
8	5.55	AT3G05545	RING/U-box superfamily protein	nucleus	no
9	5.39	AT1G07950	Surfeit locus protein 5 subunit 22	nucleus	no
10	5.36	AT2G46505	succinate dehydrogenase subunit 4	mitochondrion	YES
11	5.36	AT4G10810	hypothetical protein	nucleus	no
12	5.34	AT5G60460	Preprotein translocase Sec61-beta subunit	golgi	no
13	5.18	AT4G28060	Cytochrome c oxidase, subunit Vib family protein	mitochondrion	YES
14	5.18	AT5G07470	peptidemethionine sulfoxide reductase 3	nucleus	no
15	5.1	AT4G37830	Cytochrome c oxidase, subunit Via	mitochondrion	YES
16	4.91	AT1G22840	Cytochrome c1	mitochondrion	YES
17	4.9	AT2G45070	Preprotein translocase Sec61-beta subunit	ER	no
18	4.89	AT5G64400	CHCH domain protein	mitochondrion	YES
19	4.78	AT3G13910	hypothetical protein (DUF3511)	cytosol	no
20	4.76	AT5G67600	cysteine-rich TM module stress tolerance protein	plasma membrane	no

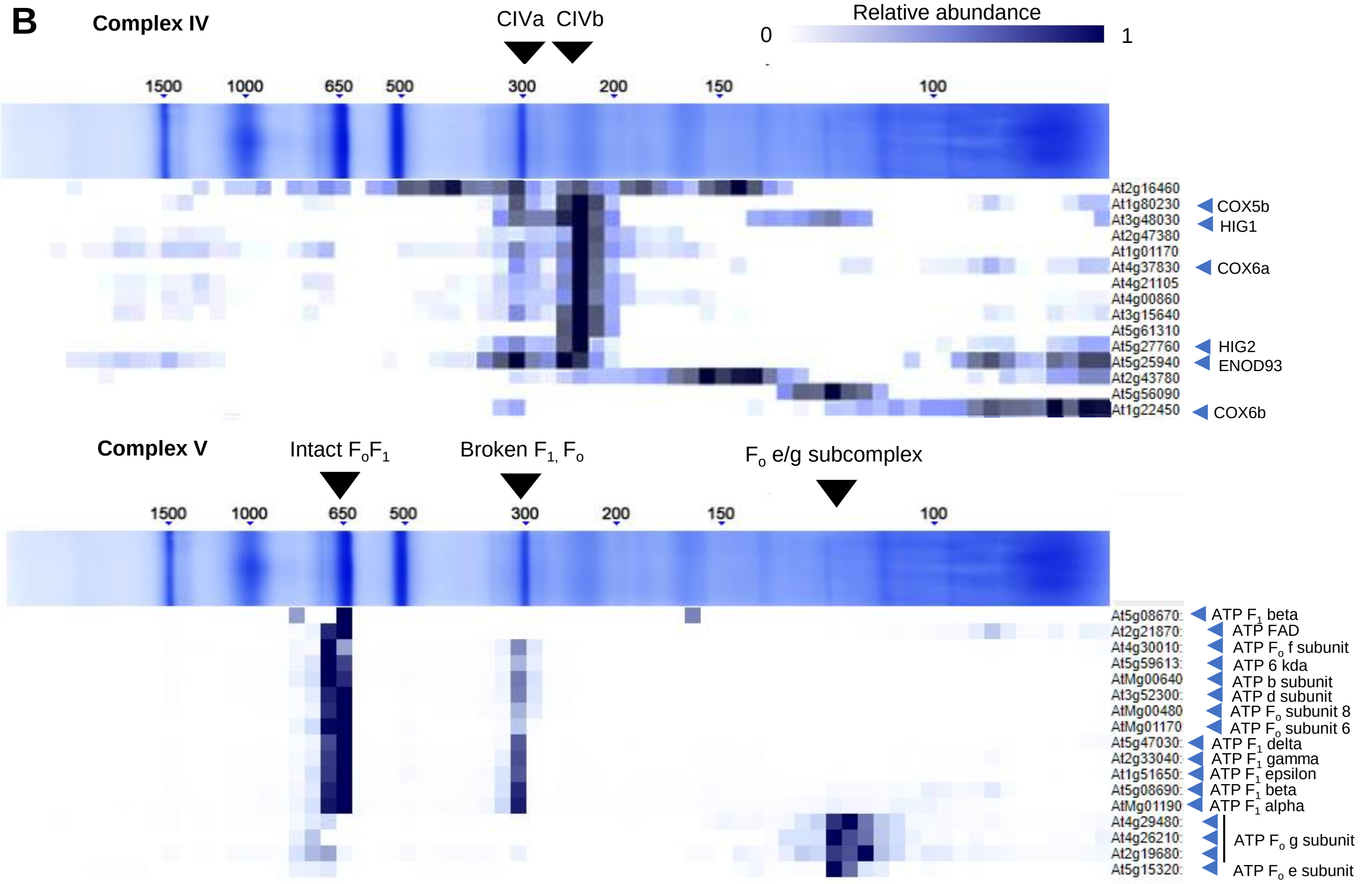

Supplemental Figure S3. Co-expression and intra-mitochondrial localisation of ENOD93. (A) The 20 genes most co-expressed with *ENOD93* in Arabidopsis from the co-expression function in ATTED-II (Obayashi et al., 2022), the predicted subcellular localization of the encoded proteins as determined by SUBAcon (Hooper et al., 2014) and their presence/absence in the Arabidopsis mitochondria complexome map (Senkler et al., 2017). Genes for subunits of cytochrome c oxidase are highlighted. (B) Reproduction of the clustering of the relative abundance of ENOD93 peptides with those of other complex IV subunits in the Arabidopsis mitochondria complexome map (Senkler et al., 2017) using complexomemap.de, and annotation of the size of the two intact versions of CIV; CIVa and CIVb (Millar et al., 2004), and the location of ENOD93, HIG1, HIG2, COX5b, COX6b and COX6a; and comparison to F_OF_1 ATP Synthase subunits analysed in the same experiment from Senkler et al (2017) (The same blue native gel picture is shown), F_1 subunits first row of arrows, F_O and stator stalk subunits in second row of arrows. (Supports Figure 2).

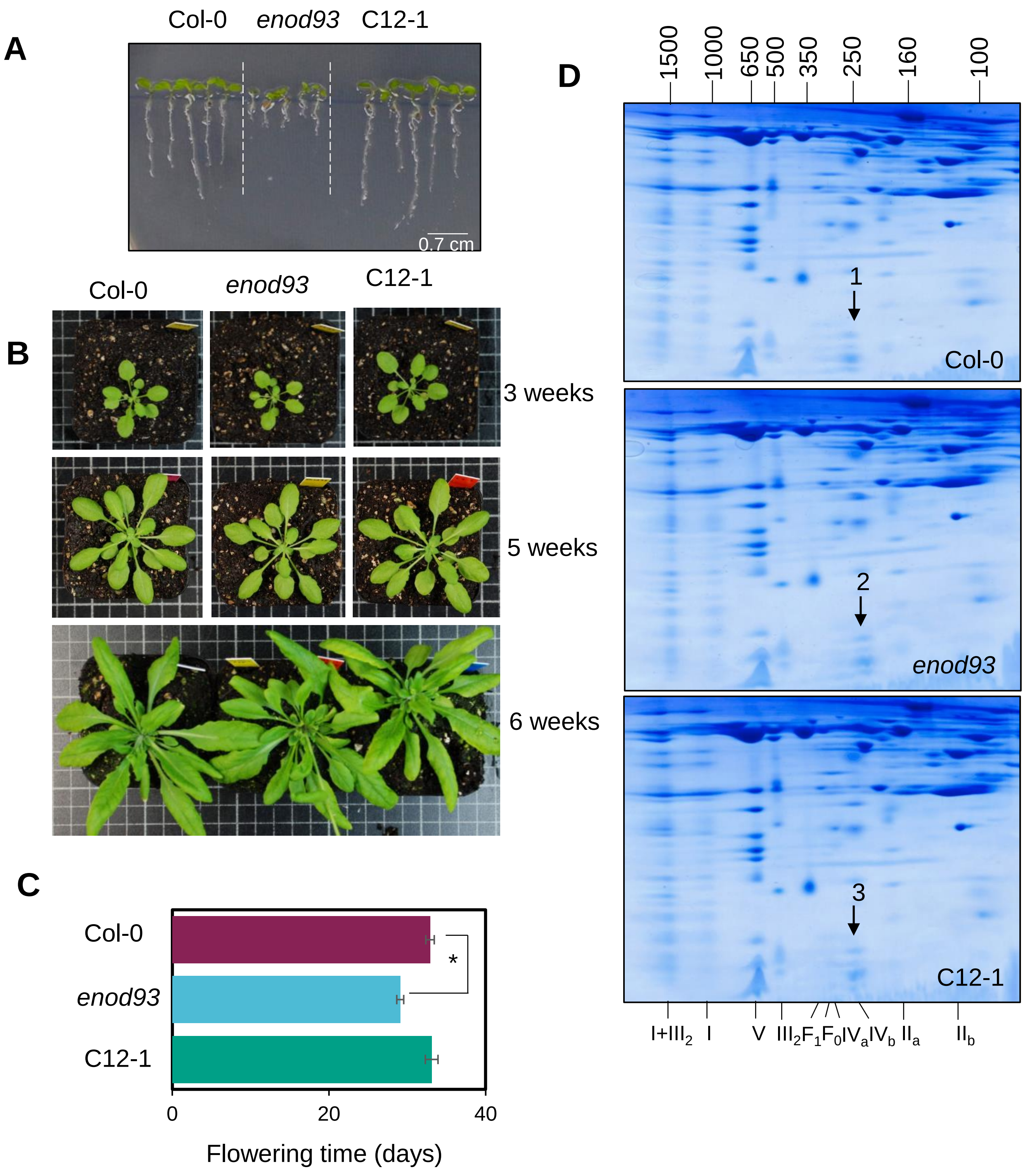

Supplemental Figure S4. Phenotype of *enod93 and* complemented line and identification of ENOD93. (A) Representative image of vertically grown 8-day-old seedlings of Col-0, *enod93* and complemented lines on an agar plate under long day condition. (B) Phenotype of Col-0, *enod93*, and C12-1 lines in 3, 5 and 6 weeks of long-day light growth conditions. (C) Flowering time of Col-0, *enod93* and C12-1. Data represents mean ± S.E. Asterisks indicate a significant change relative to Col-0 as determined by Student's t-test (*p<0.05, n=10). (D) 2D-blue-native/SDS-PAGE separation of mitochondrial proteins from the three genotypes. Roman numerals correspond to the locations of respiratory complexes, numbers are approximate molecular mass in kDa. Gels were visualised by Coomassie Blue. Location of ENOD93 containing spot is marked by arrows and verified by LC-MS/MS, with the corresponding numbers correlating with Supplementary Table S2 (Supports Figure 2).

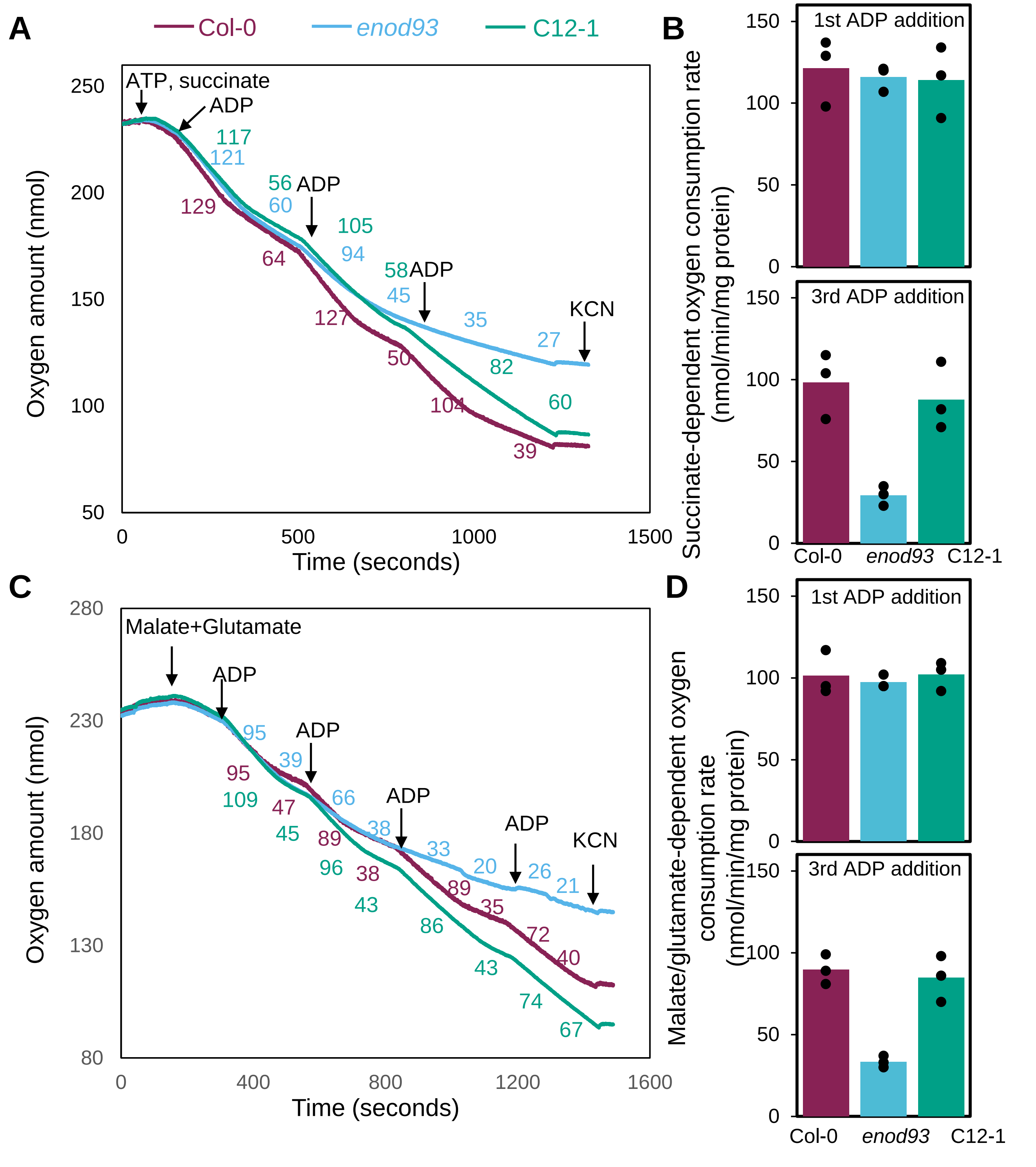

Supplemental Figure S5. Succinate- or malate/glutamate-dependent respiration by isolated *enod93* mitochondria. (A) Representative trace illustrating succinate-stimulated oxygen consumption by mitochondria purified from Col-0, *enod93* and C12-1 (a complementation line) seedlings. Oxygen consumption rates in response to the successive treatments are shown in coloured numerical values. Substrate additions to all samples are indicated by arrows with the following concentrations: 5 mM succinate, 0.5 mM ATP, 0.1 mM ADP and 1 mM KCN. (B) State III oxygen consumption rates for purified mitochondria energized with succinate in response to the first (upper panel) and third (lower panel) ADP addition as indicated in (A). Data represents mean with overlaid individual data points as dots (n = 3). (C) Representative trace illustrating malate/glutamate-stimulated oxygen consumption by mitochondria purified from Col-0, *enod93* and C12-1 seedlings. Oxygen consumption rates in response to the successive treatments are shown in coloured numerical values. Substrate additions to all samples are indicated by arrows with the following concentrations: 10 mM malate, 10 mM glutamate, 0.1 mM ADP and 1 mM KCN. (D) State III oxygen consumption rates for purified mitochondria energized with malate and glutamate in response to the first (upper panel) and third (lower panel) ADP addition as indicated in (C). Data represents mean with overlaid individual data points as dots (n = 3) (Supports Figure 3).

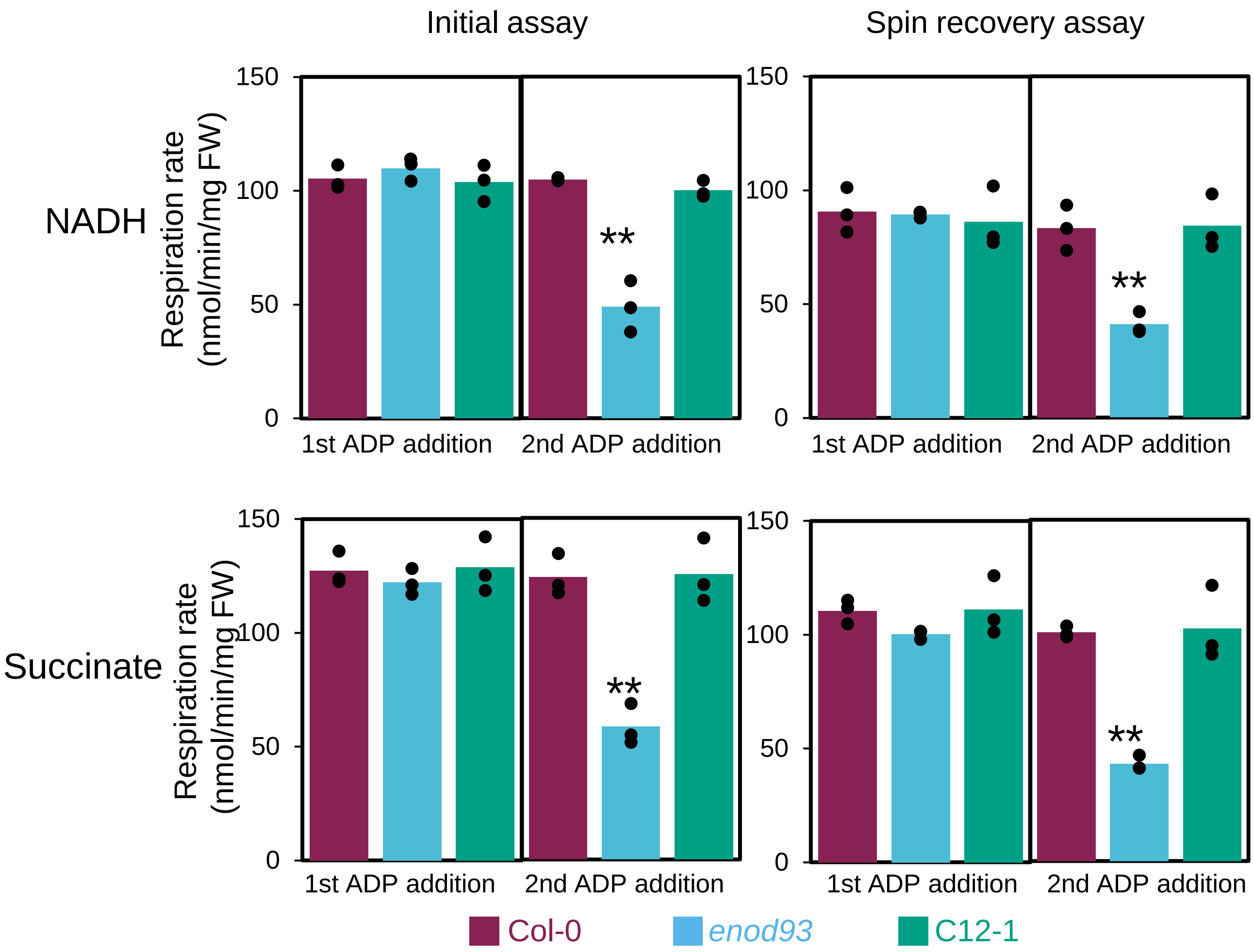

Supplemental Figure S6. Spin recovery assays of respiratory rate in isolated *enod93* mitochondria. For Initial assay**,** NADH- (upper panels) or succinate- (lower panels) dependent state 3 respiration measurements were carried out on freshly purified mitochondria. Mitochondrial fractions were collected and subjected to a few rounds of washes with respiration medium without substrates to dilute or remove substrates, products and ATP. Respiration measurements were then repeated on washed mitochondria fractions, as shown under the spin recovery assay heading. All data represents mean ± S.E. with overlaid individual data points as dots (n = 3). Asterisks indicate a significant change in Col-0 vs *enod93* and *enod93* vs C12-1 comparisons as determined by one-way ANOVA with Tukey post-hoc test (* $p < 0.05$; ** $p < 0.01$) (Supports Figure 3).

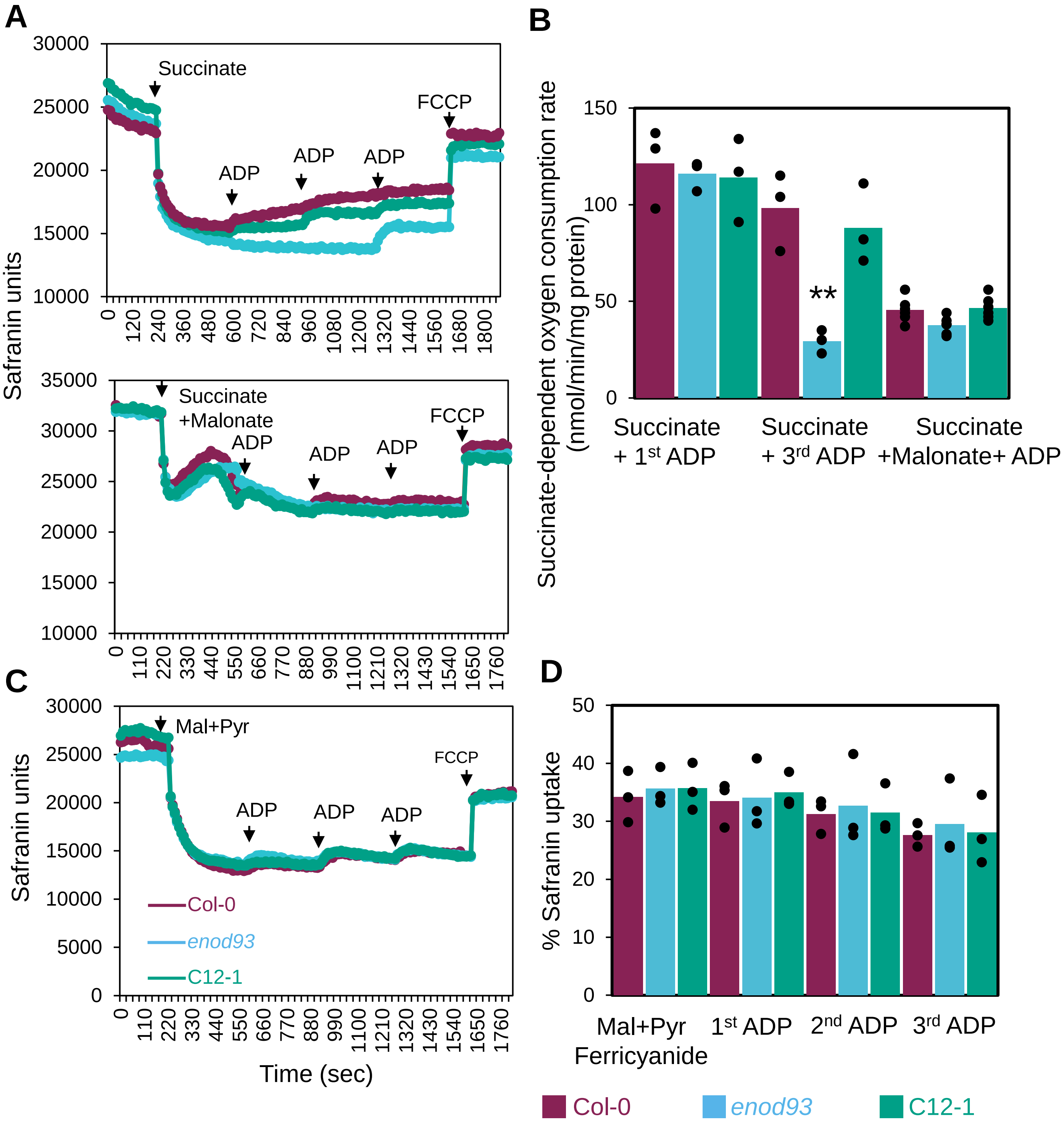

Supplemental Figure S7. Effect of lowering membrane potential and complex IV dependence based on differences between Col-0, *enod93* and complemented line mitochondrial bioenergetics. (A) Representative trace of safranin uptake (n=1) as a measure of membrane potential in mitochondria from Col-0, *enod93* and C12-1 after multiple ADP additions during succinate-stimulated respiration with/without 0.3mM malonate as a complex II inhibitor. (B) Oxygen consumption rates for purified mitochondria energized with succinate in response to ADP addition with and without 0.3 mM malonate. Data represents mean with overlaid individual data points as dots (n = 3). Asterisks indicate a significant change as determined by Student's t-test (** $p < 0.01$). (C) Representative trace of safranin uptake (n=1) as a measure of membrane potential in mitochondria from Col-0, *enod93* and C12-1 after multiple ADP additions during malate/pyruvate-stimulated respiration in the presence of 3 mM ferricyanide as an alternative oxygen acceptor to bypass cytochrome c oxidase. (D) % safranin uptake in repeated experiments. Data representing mean with overlaid individual data points as dots (n = 3). Asterisks indicate a significant change as determined by Student's t-test ($p < 0.05$)(Supports Figure 5).

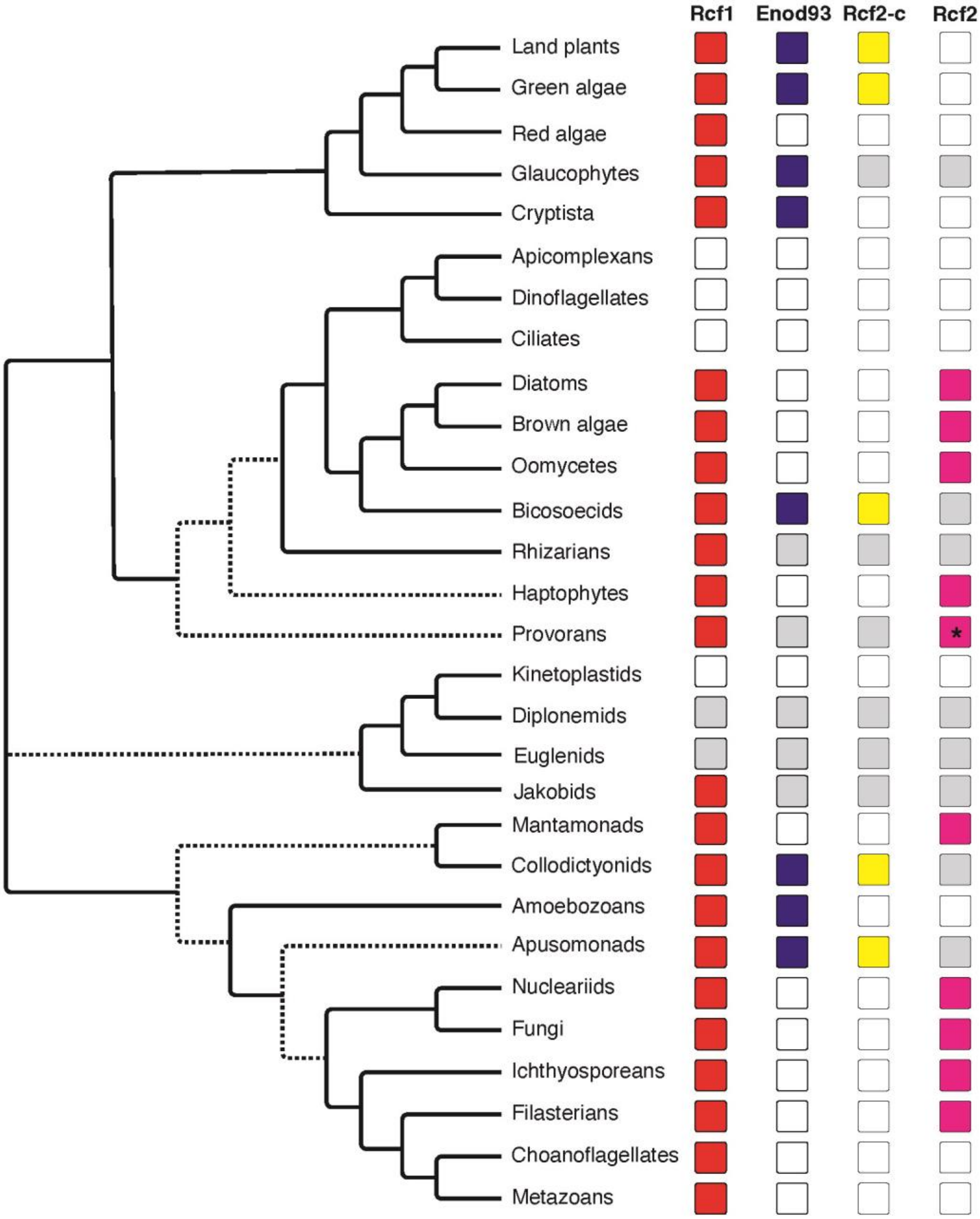

Supplemental Figure S8. A schematic of complex distribution of plant- and fungal-type Rcf2 homologs across the breadth of eukaryotes. The distribution of plant- and fungal-type Rcf2 does not precisely mirror eukaryotic phylogeny, suggesting the multiple gene fusions and/or fissions have occurred. Presence of Rcf1 (red), plant-type Rcf2 – consisting of Enod93 (blue) and Rcf2-c (yellow) – and fungal-type (magenta) Rcf2 are shown. Empty boxes indicate an inability to find homologs in listed groups. Boxes shaded light gray also indicate that homologs were not found, but that insufficient genomic resources are available to be confident of absence. Dashed lines denote tentative associations between major eukaryotic lineages. An asterisk is shown for fungal-type Rcf2 in Provorans because protein domains are fused, but in the opposite orientation of fungal Rcf2 (Supports Figure 1).

Supplementary Table S1. Identification of ENOD93 from 2D-BN/SDS-PAGE. Protein spots marked in Figure S4B were identified by LC-MS/MS. Identified proteins with at least 2 peptides and total ion score >50. The Mascot match score, number of peptides identified and the exponentially modified protein abundance index (emPAI) score is provided.

			**Col-0 (Spot 1)**			**endo93 (Stop 2)**			**Comp 12-1 (Spot 3)**		
**AGI accession**	**Description**	MW	Match Score	#pep-tide	emPAI	Match Score	#pep-tide	emPAI	Match Score	#pep-tide	emPAI
AT5G43970.1	translocase of outer membrane 22-V	10372	1566	6	7.27	1766	8	10.68	1950	7	10.71
**AT5G25940.1**	**early nodulin 93**	**12262**	**197**	**5**	**3.48**	**-**	**-**	**-**	**198**	**6**	**3.47**
AT4G30010.1	ATP-dependent RNA helicase	10432	172	7	10.47	267	7	10.4	162	7	10.42
AT5G24165.1	hypothetical protein	7751	63	2	0.58	58	4	0.58	60	2	0.58
AT5G61220.1	LYR family of Fe/S cluster biogenesis protein;	10108	60	7	7.64	206	8	7.59	58	6	5.02

Supplementary Table S2. Primer sequences used in the study

**Primers**	**Sequence (5′–3′)**
Cloning and genotyping	
AtENOD93-GFwd	GGGGACAAGTTTGTACAAAAAAGCAGGCTATG<br><br>GAAAATCGGTCAGAAATGGGTA
AtENOD93-GRev	GGGGACCACTTTGTACAAGAAAGCTGGGTTTA<br><br>TTTAGAGTCTTGCTGAACTTTCTT
AtENOD93-Fwd	ATGGAAAATCGGTCAGAAATGGGTA
AtENOD93-Rev	TTATTTAGAGTCTTGCTGAACTTTCTT
35S-Fwd	CTATCCTTCGCAAGACCCTTC
pB2GW7-Fwd	CCGTGAAGACTGGCGAACA
atenod93-LP	ATCGGTCAGAAATGGGTAACC
atenod93-RP	CGAAGAAGACTGCAACAGAGG
LBb1.3	ATTTTGCCGATTTCGGAAC
TDNA-Seq LB-R	CGAGCCGAAACTTCCAAACA
RT-qPCR	
AtENOD93-qFwd	CACAGTAGCTGCCGTTCGTA
AtENOD93-qRev	AAGAACGCAGCGATAGAAGC
Act-Fwd	TGCACCGCCAGAGAGAAAAT
Act-Rev	TGAGGGATGCAAGGATTGATC

Supplementary Table S3. MRM parameters for detecting nucleotides

Compound	Precursor ion	Product ion	RT	Collision energy
$^{13}C_5$-$^{15}N_5$-AMP	363	146	7.4	16
$^{13}C_5$-$^{15}N_5$-AMP	363	101.9	7.4	32
ADP	428	348	7.55	16
ADP	428	136	7.55	24
ATP	507.9	410	7.7	16
ATP	507.9	348	7.7	16
ATP	507.9	136	7.7	36
